# Experimentally tracing water molecules and mapping high-resolution liquid structure pictures for promising water-in-salt/ionic liquid electrolytes

**DOI:** 10.1093/nsr/nwag125

**Published:** 2026-03-06

**Authors:** Le Yu, Sijun Wang, Jing Huang, Xuanyu Zeng, Xiaoyan Zhou, Zhiqiang Wang, Junqing Chen, Jiliang Liu, Chaoji Chen

**Affiliations:** School of Resource and Environmental Sciences, Hubei Biomass-Resource Chemistry and Environmental Biotechnology Key Laboratory, Wuhan University, Wuhan 430079, China; School of Resource and Environmental Sciences, Hubei Biomass-Resource Chemistry and Environmental Biotechnology Key Laboratory, Wuhan University, Wuhan 430079, China; School of Resource and Environmental Sciences, Hubei Biomass-Resource Chemistry and Environmental Biotechnology Key Laboratory, Wuhan University, Wuhan 430079, China; School of Resource and Environmental Sciences, Hubei Biomass-Resource Chemistry and Environmental Biotechnology Key Laboratory, Wuhan University, Wuhan 430079, China; School of Resource and Environmental Sciences, Hubei Biomass-Resource Chemistry and Environmental Biotechnology Key Laboratory, Wuhan University, Wuhan 430079, China; School of Science, Hubei University of Technology, Wuhan 430070, China; School of Resource and Environmental Sciences, Hubei Biomass-Resource Chemistry and Environmental Biotechnology Key Laboratory, Wuhan University, Wuhan 430079, China; School of Resource and Environmental Sciences, Hubei Biomass-Resource Chemistry and Environmental Biotechnology Key Laboratory, Wuhan University, Wuhan 430079, China; European Synchrotron Radiation Facility (ESRF), Grenoble 38000, France; School of Resource and Environmental Sciences, Hubei Biomass-Resource Chemistry and Environmental Biotechnology Key Laboratory, Wuhan University, Wuhan 430079, China

**Keywords:** water-in-salt electrolytes, liquid structure, ionic liquids, solvation sheath, electrochemistry

## Abstract

Maximizing salt concentration to eliminate the active bulk-like water is the underlying logic of the ‘water-in-salt’ (WiS) strategy in advancing high-voltage aqueous battery chemistries, whereas salts with solubility capable of reaching the WiS regime (typically water-to-salt ratio < 5) are not commonplace. Here, we showcase that, by incorporating certain ionic liquids (ILs), any of the common Zn salts can be formulated into Zn electrolytes that fall into the WiS regime. As a proof-of-concept study, focusing on a ternary Wi(S/IL) system that features a large single-phase liquid region, we demonstrate that the complementary use of infrared/NMR/Raman spectroscopy, electrospray ionization mass spectrometry and synchrotron small-angle X-ray scattering succeed in precisely depicting how the local environment of H_2_O molecules, primary/secondary solvation sheaths of Zn^2+^, and other long-range molecular arrangements are structured. Further studies of electrochemical property, Zn cycling and electrolyte/Zn interphasial chemistry performed on a Wi(S/IL) electrolyte with optimal composition reveal that fully eliminating bulk-like water and constructing a weakly bonded anionic primary solvation sheath can promise a 4-V class electrolyte operation window and decent Zn plating/stripping reversibility of 99.7% over long-term cycling. This work implies a revolution of WiS strategy and, more importantly, a new paradigm for experimentally studying the liquid structures of aqueous electrolytes.

## Teaser

We develop an effective workflow to uncover the liquid structure and performance of high-concentration electrolytes for advanced aqueous zinc metal batteries.

## INTRODUCTION

The discovery of ‘water-in-salt’ (WiS, i.e. ultrahigh salt concentration and proportion) electrolytes with a significantly expanded electrochemical stability window (ESW) approaching 3 V brings us a big step closer to realizing practical aqueous batteries [1]. The underlying logic of the WiS strategy is to minimize H_2_O molecules in the solvation sheath of the working ions (such as Li^+^ ions for lithium batteries) and concurrently disrupt hydrogen bonding interactions among H_2_O molecules. In such aqueous systems, water activity and water-induced hydrogen evolution reaction (HER) can be substantially reduced, enabling much wider ESWs of the electrolytes toward high-voltage aqueous battery chemistries [2,3]. Apparently, a prerequisite for developing WiS electrolytes is that the salts used for providing working ions should have high solubility with high concentrations in water [4]. However, while the discovery of a lithium bis(trifluoromethanesulfonyl)imide (LiTFSI)-based WiS aqueous electrolyte—21 m LiTFSI (where m represents mol kg^−1^)—has revolutionized the research of aqueous rechargeable lithium batteries, the application of the WiS strategy in aqueous electrolytes for Zn batteries has been fundamentally plagued by limited solubility of the prevailingly used Zn salts in water, e.g. 1.6 mol kg^−1^ for Zn(Ac)_2_ (Ac = acetate), 3.6 mol kg^−1^ for ZnSO_4_, and 6.3 mol kg^−1^ for Zn(OTf)_2_ (OTf = trifluoromethanesulfonate) [3,5]. The only exception is ZnCl_2_, which has a considerably high solubility of 30 mol kg^−1^ in water and has been proved to enable high Zn plating/stripping reversibility; however, its nature of poor oxidation stability and high corrosiveness toward battery components are well known yet very tricky to the aqueous Zn battery research community [6,7]. An early effort of WiS electrolytes for Zn batteries, made by Wang and colleagues [8], is partially substituting LiTFSI with Zn(TFSI)_2_ to obtain a variation of 21 m LiTFSI, i.e. 1 m Zn(TFSI)_2_ + 20 m LiTFSI. Despite a reasonably high ionic conductivity of ∼5 mS cm^−1^ among WiS electrolytes, the rate capability of Zn batteries using such an electrolyte is far from practical application due to the very low mole fraction of Zn^2+^ as carriers of ionic current, which is susceptible to concentration polarization. More recently, using the hydrotropic effect of suitable supporting salts to break the solubility limits of single Zn salt aqueous electrolytes has led to the findings of binary salt/ternary salt WiS systems. For example, the Zn(Ac)_2_–KAc water-in-bisalt (WiBS) electrolyte system, proposed by Chen *et al.* [9] and creatively reshaped by Gomez Vazquez *et al.* and Dong *et al.* [3,5], shows a wide tunability of Zn salt concentration and water-to-salt ratio and can enable high Zn plating/stripping efficiency of 99.6%–99.8%. However, summarizing all the achievements of the WIS strategy for Zn batteries, it is found that Zn(Ac)_2_ or ZnCl_2_ is still a must-have choice of Zn salt except for the cases where highly soluble but non-active alkali metal salts such as LiTFSI are incorporated as the primary salt [3,5]. This may explain why the development of WiS electrolytes for Zn batteries lags far behind that of aqueous alkali-ion batteries.

**Figure 1.**
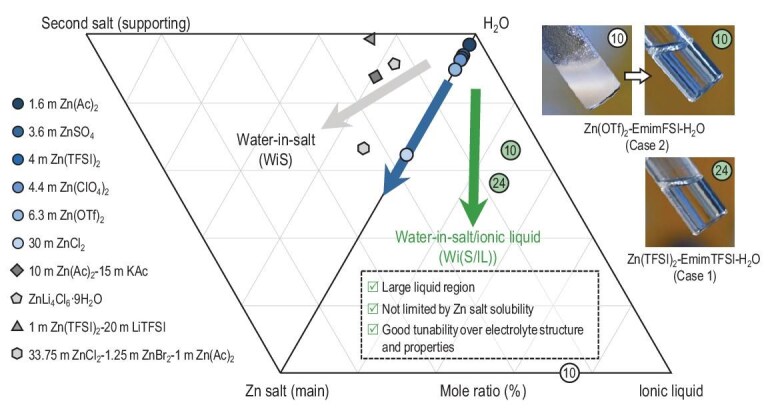
Schematic of a combined ternary phase diagram (in mole fraction) of WiS and Wi(S/IL) Zn electrolytes. The water-to-salt ratio afforded by one single Zn salt can hardly reach the WiS electrolyte regime; meanwhile, the choice of a second salt capable of reducing the water-to-salt ratio is also limited. The data (gray polygons) shown in the figure are taken from Dong *et al.* [5] (10 m Zn(Ac)_2_ + 15 m KAc), Wang *et al.* [8] (1 m Zn(TFSI)_2_ + 20 m LiTFSI), Cai *et al.* [19] (ZnCl_4_Cl_6_·9H_2_O) and Yang *et al.* [20] [33.75 m ZnCl_2_–1.25 m ZnCl_2_–1 m Zn(Ac)_2_]. The digital photos present two of a host of experimentally obtained Wi(S/IL) electrolytes that feature a low water-to-salt ratio, with corresponding mole fractions (green cycles) shown in the figure. The number inside is related to the selection of Zn salt and IL. For example, the circles labeled ‘10’ indicate that the corresponding electrolytes belong to ***E*10** (see Fig. S1 for more details).

Motivated by the advantageous non-volatility, high thermal stability and sufficient electrochemical stability, ionic liquids (ILs), also referred to as room-temperature or low-temperature molten salts [10], have been used as co-solvents of aqueous electrolytes toward high-voltage aqueous battery chemistries [11–14], but research in this field is still in an infancy stage. Even less research effort has been devoted to how these various ILs regulate the microstructure and macroscopic physicochemical properties of aqueous electrolytes [15,16]. From our perspective, at least the following two evident advantages related to ILs would drive researchers to rethink the overlooked academic importance and potential application value of the water-in-(salt/IL) [Wi(S/IL)] aqueous electrolyte system: (i) in cases that the IL can dissolve the working ion-containing salt and concurrently the resulting solution being miscible with water, the IL plays the roles of both solvent and supporting salt. This probably generates the hydrotropic effect to enable ultrahigh salt concentrations of up to 60 m [12,17]; (ii) alternatively, the IL can not dissolve the salt but is miscible with the aqueous solution of the salt. It can here be considered as an electrochemically inert supporting salt capable of reduction of water activity and thus water-related parasitic reactions [18]. A proven example of such a mechanism is that 1-ethyl-3-methylimidazolium bis(fluorosulfonyl)amide (EmimFSI) is incapable of dissolving zinc trifluoromethanesulfonate [Zn(OTf)_2_] but can significantly stabilize the conventional Zn(OTf)_2_ aqueous electrolyte by occupying the interspaces of primary Zn^2+^ solvates [18]. These beneficial effects specific to ILs would open a vast design space for Wi(S/IL) strategy towards high-demanding aqueous electrolytes, as Fig. 1 indicates [5,8,19,20].

In this work, we first consolidate this view by showing that 16 combinations generated by water, five popularly used Zn salts, and five commercial ILs (namely 25 possible combinations, numbered ***E*1** to ***E*25**) possess significant single-phase liquid regions covering the low water-to-salt regime, which all formally belong to the Wi(S/IL) system and have application potential as Zn electrolytes (see Fig. S1 and Note S1 for details). Of all these Wi(S/IL) systems, the one composed of Zn(TFSI)_2_, EmimTFSI and H_2_O (***E*24**) is chosen for in-depth liquid structure–electrochemical property–Zn anode chemistry research due to the following factors: primarily, our preliminary experimental investigation shows that the ternary solubility phase diagram of Zn(TFSI)_2_–EmimTFSI–H_2_O has a larger single-phase liquid region (indicative of the broad composition fraction tunability), which is critical for exploring the boundaries of various properties and achieving the best overall performance; secondly, the ionic components of this electrolyte system are quite similar to the well-known WiS electrolyte of 1 m Zn(TFSI)_2_ + 20 m LiTFSI. Fruitful research results of this electrolyte are of great reference significance for conducting a comparative study [21–26]. Using the spontaneous H/D exchange between deuterated water (D_2_O) and C(2)–H on Emim [27–29], we introduce the O–D stretch of HOD molecules as an incisive probe to non-destructively monitor the local environment of water and successfully delineate water’s evolutionary trajectory in response to the reduction of water concentration. By combining proton nuclear magnetic resonance (^1^H NMR), Raman, electrospray ionization mass spectrometry (ESI-MS) and synchrotron small-angle X-ray scattering (SAXS) characterizations, detailed liquid structure pictures of the Zn(TFSI)_2_–EmimTFSI–H_2_O Wi(S/IL) electrolyte and representative dilute and WiS electrolytes of 1 m Zn(TFSI)_2_ and 1 m Zn(TFSI)_2_ + 20 m LiTFSI were mapped. Further electrochemical properties and Zn cycling performance study reveal that the Wi(S/IL) can afford a 3.8 V operation window, a high Zn plating/stripping reversibility averaging 99.7%, and ultrastable interphasial chemistry indicated by a 1-year Zn cell cycling lifespan (>10 000 cycles), all of which are experimentally identified to correlate with its liquid structure.

## RESULTS AND DISCUSSION

### Correlating IR spectroscopic response and molecular state of water in Wi(S/IL)

**Figure 2.**
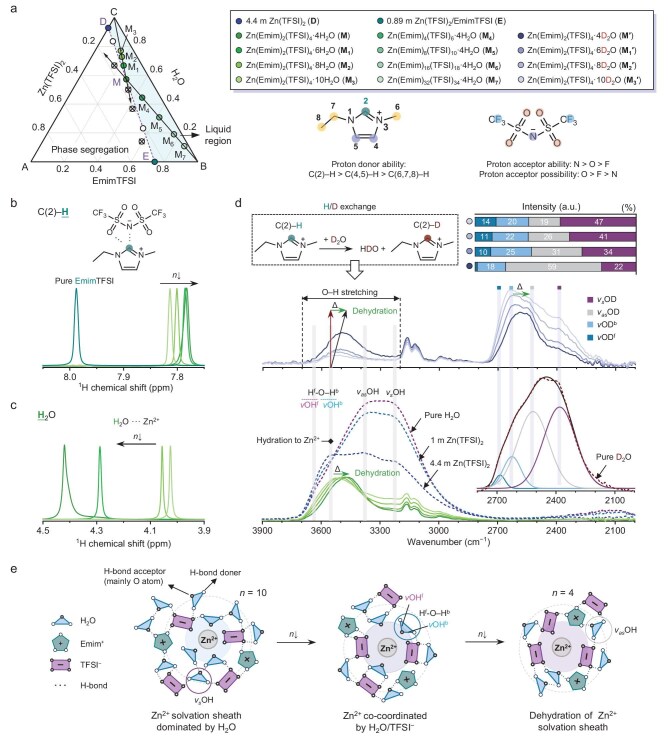
Evolution of the local environment of Zn^2+^ ions and H_2_O molecules. (a) A rough solubility diagram (in mole fraction) of Zn(TFSI)_2_–EmimTFSI–H_2_O ternary electrolyte system obtained at 303 K. Closed colored circles correspond to experimental electrolyte samples of interest, open black circles represent the electrolytes with corresponding formulations that are single-phase liquid, while those marked with a cross represent solid–liquid mixtures. The structure formula of EmimTFSI and the atomic numbering scheme of the Emim cation. A qualitative comparison of the proton donor ability of H atoms on Emim^+^ and the proton acceptor ability/possibility of N, O and F atoms on TFSI^−^ is given by combining the previous experimental/theoretical findings [30–32]. Note that the colors of legend entries included in (a) apply to all panels. ^1^H NMR chemical shift of (b) C(2)–H and (c) H_2_O for Zn(Emim)_2_(TFSI)_4_·*n*H_2_O (*n* = 4, 6, 8, 10) to that of C(2)–H for pure EmimTFSI is included. Both are normalized by the maximum value within the set. The bottom panel of (d) shows the FTIR spectra of Zn(Emim)_2_(TFSI)_4_·*n*H_2_O (*n* = 4, 6, 8, 10), pure water, 1 m Zn(TFSI)_2_ and 4.4 m Zn(TFSI)_2_. The middle panel shows the FTIR spectra of Zn(Emim)_2_(TFSI)_4_·*n*D_2_O (*n* = 4, 6, 8, 10), with the proportions of four sub-bands inside the O–D stretching band presented in the bar chart. The bands are interpreted, deconvoluted and assigned by referring to the existing knowledge of the O–D stretching for pure D_2_O [inset of (d)]. Fitting parameters and results of the O–D stretching band of all D_2_O-based electrolytes are shown in Fig. S4 and Table S2. (e) Cartoon illustrating the evolution of the local environment of Zn^2+^ as *n* decreases from 10 to 4.

We start by producing the simplified Zn(TFSI)_2_–EmimTFSI–H_2_O ternary solubility diagram at 30°C, as shown in Fig. 2a (for details see Note S2). From the single-phase liquid region, two groups for a total of eight electrolyte samples (**M**–**M_7_** ∈ ***E*24**) were selected for further experimental investigation (see Table S1 and Note S3 for the exact electrolyte compositions and general considerations about electrolyte selection). Water governs the molecular organization of WiS electrolytes by regulating the intermolecular hydrogen bonding motifs and solvation structure of metal cations, more significantly in the investigated Wi(S/IL) electrolytes, where water can heavily participate in forming an array of hydrogen bonding interactions with the IL components. Conversely, it means that by deciphering the hydrogen bonding chemistries, we can reasonably depict the liquid structure of such systems. To this end, ^1^H NMR and attenuated total reflectance Fourier-transform infrared spectroscopy (FTIR) investigations of electrolytes **M**–**M_7_** and reference samples (**B** for pure EmimTFSI, **C** for pure water and **D** for 4.4 m Zn(TFSI)_2_) were performed (see Figs S2 and S3 for the full NMR and FTIR spectra). As the ^1^H NMR spectrum of **B** shows (Fig. S2), the chemical shift values of H atoms on the imidazolium ring and side alkyl chain are indicative of their acidities (hydrogen bond capability) in an order of C(2)–H > C(4,5)–H > C(6,7,8)–H (see the atomic numbering scheme of Emim^+^ in Fig. 2a) [30–32]. With the introduction of Zn(TFSI)_2_ and water, all the ^1^H NMR resonances belonging to Emim^+^ gradually shift to the high field direction as a result of an enhancement of electron density around the hydrogen nucleus (shielding effect). This is reasonably related to the strong hydrogen bonding existing in EmimTFSI (possible interactions include C(2)/C(4,5)–H···N/O/F(TFSI)) are being replaced by the newly formed hydrogen bonding interactions between Emim^+^ and water [mainly C(2)–H···O(water)] [17,18]. The shift difference (Δδ^1^H) of C(2)–H and C(4,5)–H is more significant than that of C(6,7,8)–H, as the latter ones are less prone to hydrogen bond formation. Focusing on the most influential hydrogen bonding site of C(2)–H (Fig. 2b), the Δδ^1^H increases monotonically with the decrease of *n* value for Zn(Emim)_2_(TFSI)_4_·*n*H_2_O (10 → 8 → 6 → 4) from 0.174 to 0.205 ppm, suggesting a puzzling process that the Emim^+^–TFSI^−^ interactions are increasingly being disrupted by water in cases of low water concentrations. For the group of electrolytes with a fixed H_2_O/Zn^2+^ stoichiometric ratio of 4 and increasing *x* value (2 → 4 → 8 → 16 → 32), the Δδ^1^H drops from 0.205 to 0.011 ppm as the Emim^+^–TFSI^−^ interaction gradually dominates again. Further examining the ^1^H NMR resonance of H_2_O in Zn(Emim)*_x_*(TFSI)*_x_*_+2_·*n*H_2_O (Fig. 2c), a continuous downfield shift from 4.03 to 4.42 ppm due to the reduction in surrounding electron density of the hydrogen nucleus (deshielding effect) is observed with decreasing *n* value, which along with the shielding of all hydrogen atoms on the Emim^+^ cation, hint at the withdrawal of water initially residing in the solvation sheath of Zn^2+^ (dehydration) to form hydrogen bonds with Emim^+^ and TFSI^−^. The ^1^H NMR chemical shift of H_2_O stabilizes within 4.40–4.56 ppm regardless of the *x* value, signifying that electrolyte **M** is also a turning point where the dehydration of Zn^2+^ solvates has reached the limit.

To depict the liquid structure picture of Zn(Emim)*_x_*(TFSI)*_x_*_+2_·*n*H_2_O closer to reality, deeper questions to be answered are the specific locations and intermolecular interactions of these H_2_O molecules disengaged from the Zn^2+^ solvation sheath. FTIR spectra of water’s O–H stretching vibration modes, appearing in the 3000–3800 cm^−1^ region, are very sensitive in reflecting the bonding states of water, which may provide additional molecular-level details regarding the local environment around H_2_O molecules (see Note S4 for interpretations of the O–H and O–D bending vibration modes) [33]. Somewhat consistent with the ^1^H NMR results, the O–H stretching vibration mode does not show any shift with increasing *x* value (Fig. S3), which is only able to indicate that water in Zn(Emim)*_x_*(TFSI)*_x_*_+2_·4H_2_O (*x* = 2, 4, 8, 16, 32) features a similar bonding environment. In sharp contrast, observing the set of FTIR spectra for Zn(Emim)_2_(TFSI)_4_·*n*H_2_O (*n* = 4, 6, 8, 10) (bottom panel of Fig. 2d), it was found that the O–H stretching band, with a peak intensity achieved at 3527 cm^−1^ for *n* = 10, experiences continuous redshifts gradually to 3461 cm^−1^ for *n* = 4. This result is very informative despite being contradictory to the general understanding that the low-frequency regime of the O–H stretching band is typically related to fully hydrogen-bonded H_2_O molecules, which are more commonly seen in water-rich systems. To interpret this ‘anomalous’ shift, a proper deconvolution of the O–H stretching band of water is warranted (water O–H stretching band could be deconvoluted into 3 to 6 sub-bands depending on the model used, none of which is yet able to perfectly describe the unique molecular structure of water) [34].

Herein, the spectra of pure water and aqueous solutions of 1 mol kg^−1^ Zn(TFSI)_2_ and 4.4 mol kg^−1^ Zn(TFSI)_2_ (**D**) were included to indicate the most suitable model for the assignment of sub-bands inside the O–H stretching band. Compared to that of pure water, the O–H stretching band of water in 1 mol kg^−1^ Zn(TFSI)_2_ (dashed purple line) features a distinguishable shoulder at 3540 cm^−1^. Meanwhile, the band in the top region is slightly split into two sub-bands centered at 3384 and 3225 cm^−1^. Despite having differences in band assignments of the proven models, it is barely controversial that these two sub-bands are respectively assigned to the asymmetrical and symmetrical O–H stretching (denoted as ν_as_OH and ν_s_OH) of H_2_O molecules that are fully hydrogen-bonded as the previous text mentioned [34,35], and the ν_s_OH stretching only originates from H_2_O molecules that are tetrahedrally bonded (also know as bulk-like water) [35]. With Zn salt concentration reaching 4.4 mol kg^−1^, the band intensity at 3540 cm^−1^ experiences a further significant increase, for which we reasonably relate this sub-band to one of the stretching vibration modes of water in hydrated Zn^2+^ ions. Additionally, almost all the existing models suggest that, at the higher end of the spectra, specifically around 3620 cm^−1^, there exists a weak band generally attributed to the contribution of ‘quasi-free’ O–H stretching [34,36]. This sub-band is herein found to increase gradually with the Zn salt concentration, following a similar trend with the band at 3540 cm^−1^. This drives us to believe that in conventional Zn aqueous electrolytes, the ‘quasi-free’ O–H stretching is exactly the other kind of stretching vibration mode of water in hydrated Zn^2+^ ions. Historically, the contributions of these two stretching vibration modes together have been attributed to the asymmetrically bonded H_2_O molecules (alternatively referred to as ‘weak hydrogen bonding’ in most aqueous electrolyte research) with a structural formula of H^f^-O–H^b^, where the superscript b indicates the OH oscillator is hydrogen-bonded to another molecule (denoted as OH^b^) while f represents that the OH oscillator is free of hydrogen bonding (denoted as OH^f^) [5,8,37,38].

By combining previously developed band assignment knowledge and our experimental insights into the origins of different water O–H stretching modes, a possible mechanism capable of bridging the knowledge gap from the O–H stretching band assignment of pure water to that of Zn aqueous electrolytes is that the H_2_O molecules in the solvation sheath of Zn^2+^ ions are typically asymmetrically bonded to generate two different stretching modes, νOH^f^-Zn^2+^ at the higher end of the O–H stretching band and νOH^b^-Zn^2+^ at the relatively lower wavenumber (stretching modes of such OH oscillators are highlighted by adding a suffix ‘-Zn^2+^’ hereafter). With this proposed mechanism, we additionally rationalize our interpretation that in Zn(Emim)_2_(TFSI)_4_·*n*H_2_O, it is the dehydration of Zn^2+^ solvates that causes the anomalous redshift of water O–H stretching with decreasing water concentration. Now, it is sufficiently reasonable to derive a model that deconvolutes the water O–H stretching band into four deconvolved components with interpretations customized to Zn aqueous electrolytes, namely ν_s_OH at ∼3225 cm^−1^ arising from tetrahedrally bonded bulk-like water, ν_as_OH at ∼3384 cm^−1^ from water fully hydrogen-bonded, νOH^b^-Zn^2+^ at ∼3540 cm^−1^ from the hydrogen-bonded OH oscillator, and νOH^f^-Zn^2+^ at ∼3620 cm^−1^ for the non-hydrogen-bonded OH oscillator. These four stretching modes can be divided into two subgroups. One contains ν_s_OH and ν_as_OH originating from water outside the primary Zn^2+^ solvation sheath, the νOH^b^-Zn^2+^ and νOH^f^-Zn^2+^ as the second group only relates to water coordinated with Zn^2+^ ions.

Common water was replaced with D_2_O to exclude the overlap issue of O–H and C–H stretching vibration regions for more precisely quantifying the contributions of the four component stretching modes, with results shown in the top panel of Fig. 2d (see Note S5 for details). Combining the additional clues provided by the spontaneous H/D exchange reaction between D_2_O and hydrogen bond donors on Emim^+^ [27,28], we can now conclusively identify that the H_2_O molecules escape from the Zn^2+^ solvation sheath and finally end up being multiply bonded to Emim^+^ and TFSI^−^ as water concentration decreases (*n* = 10 → 4 for electrolytes **M_3_** → **M**), yielding a TFSI^−^-dominated primary Zn^2+^ solvation sheath in electrolyte **M** as schematically depicted in Fig. 2e (see Note S6 for details); despite progressive dilution by EmimTFSI (*x* = 2 → 32 for electrolytes **M** → **M_4_** → **M_7_**), clustering of H_2_O molecules outside the primary Zn^2+^ solvation sheath intensifies (Fig. S5; for details see Note S6). This evolution trend of water environments is further corroborated by molecular dynamics (MD) simulations across all these eight electrolytes (Figs S6 and S7; for details see Note S7). Additionally, such anion-dominated solvation structures are very different from the extensively studied WiS and dilute aqueous electrolytes in which, typically, H_2_O molecules preferentially occupy the primary solvation sheath regardless of the water-to-salt ratio [8], while the underlying mechanism for these discrepancies remains to be elucidated.

### Mapping liquid structure pictures

**Figure 3.**
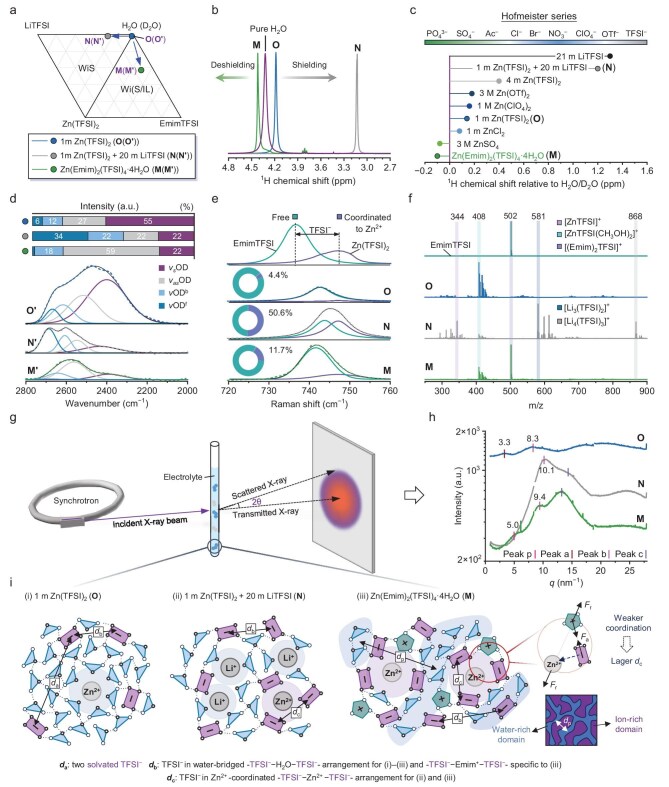
Combined spectral analysis of the microscopic liquid structure of different electrolytes. (a) Ternary phase diagram showing the compositions of three representative electrolytes of dilute aqueous electrolyte **O**, WiS electrolyte **N** and Wi(S/IL) electrolyte **M**. The legend entries included in (a) apply to all panels. (b) ^1^H NMR chemical shift of H_2_O in electrolytes, normalized by the maximum value within the set. The resonance signal of C(7)–H for electrolyte M is omitted for clarity. (c) A summary of the ^1^H chemical shift of H_2_O in our investigated electrolytes and several benchmark aqueous electrolytes (relative to that of pure water). The relative chemical shift is found to correlate with the Hofmeister series. (d) Deconvolution results and assignments of O–D stretching band in FTIR spectra of electrolytes **O′, N′** and **M′**. (e) Raman spectra in the 720–760 cm^−1^ region corresponding to the S–N–S bending vibrations in TFSI^−^. The bands are interpreted, deconvoluted and assigned based on the S–N–S bending vibrations of pure EmimTFSI and pure Zn(TFSI)_2_. (f) ESI-MS of electrolytes in positive mode. (g) Schematic of synchrotron SAXS measurement of electrolytes. (h) SAXS profiles of electrolytes. (i) Schematic illustrations of the liquid structure of electrolytes. Possible molecular arrangements and nanoscale heterogeneity that account for the observed characteristic peaks in the SAXS profiles are given.

A baseline dilute electrolyte of 1 m Zn(TFSI)_2_ (labeled as **O**) and a benchmark WiS electrolyte of 1 m Zn(TFSI)_2_ + 20 m LiTFSI (labeled as **N**) are included to conduct a comparative study for further elucidating the origin of the atypical liquid structure of Zn(Emim)_2_(TFSI)_4_·4H_2_O [a Wi(S/IL) electrolyte of interest] (Fig. 3a). To begin with, the local environment of water in electrolytes **O, N** and **M** was investigated using NMR and ATR-FTIR. As seen in Fig. 3b, in comparison with pure H_2_O, an upfield peak shift by 0.14 ppm (Δδ^1^H) is observed for electrolyte **O**. This value dramatically increases to 1.2 ppm upon the introduction of 20 m LiTFSI (electrolyte **N**), indicating the significantly enhanced shielding effect to the hydrogen nuclei of H_2_O molecules. By summarizing the relative chemical shift values of the water proton in some popularly used Zn aqueous electrolytes and those investigated in this work [7,18,22,39–41], we found that the variation trend of Δδ^1^H basically follows the anion Hofmeister series [42,43] with salt concentration as an effect enhancer (Fig. 3c; for details see Note S8). Emim^+^ functions as a strong kosmotrope to form hydrogen bonds with H_2_O molecules, freeing H_2_O molecules from the Zn^2+^ solvation sheath. This interpretation is in good agreement with our FTIR analysis results of Fig. 2d.

Further analyzing the deconvolution results of water O–D stretching bands (Fig. 3d), we found significantly different contributions of the component stretching mode for electrolytes **N** and **M**. Of note is the exceptionally high proportion of 56% for D^f^-O–D^b^ (specifically 34% for νOD^f^) of electrolyte **N**, which is in sharp contrast to that of electrolyte **M**. It is very encouraging that based on our proposed four-component model for assigning and interpreting the O–D stretching band in aqueous electrolytes, the FTIR results align well with both MD simulations and existing literature reports [8,44,45]: in WiS electrolyte **N**, most H_2_O molecules coordinate with Li⁺ while TFSI⁻ dominates the Zn^2+^ primary solvation sheath; in dilute electrolyte **O**, Zn^2+^ primarily coordinates with six H_2_O molecules—a well-established consensus here quantitatively validated by our MD simulations (Fig. S8).

Another aspect to be considered for electrolyte use is the solvation behavior of Zn^2+^. From previous discussions, it is known that Zn^2+^ ions in both electrolytes of **M** and **N** are mainly surrounded by TFSI^−^; in this case, Zn^2+^···TFSI^−^ coordination strength would play the decisive role in a host of electrochemical properties. Raman spectra in the wavenumber region of 720–760 cm^−1^, attributable to the S–N–S bending mode (*δ*_S–N–S_) in TFSI^−^, are sensitive in reflecting the Zn^2+^ (Li^+^)···TFSI^−^ coordination state [1,46,47]. We deconvoluted the *δ*_S–N–S_ bands of electrolytes **O, N** and **M** into two component bands, respectively representing free TFSI^−^ at 742 cm^−1^ (namely non-coordinated, indicated by pure EmimTFSI) and coordinated TFSI^−^ at 747 cm^−1^ [indicated by pure Zn(TFSI)_2_] (Fig. 3e). As shown, in electrolyte **O**, the contribution of coordinated TFSI^−^ is nearly negligible as an anion in a dilute aqueous electrolyte is exclusively solvated by water. Half of the TFSI^−^ in electrolyte **N** are coordinated due to a combined effect of strong Zn^2+^···TFSI^−^ coordination and high population of Li^+^···TFSI^−^ interaction despite being much weaker. The TFSI^−^ number in the Zn^2+^-solvation sheath of electrolyte **M**, estimated by multiplying the TFSI^−^/Zn^2+^ ratio with the proportion of coordinated TFSI^−^, is much lower than that of a typical Zn^2+^···TFSI^−^ solvation complex, which is about 0.47 versus 3. This is tentatively reasoned as TFSI^−^ is only weakly coordinated to Zn^2+^.

ESI-MS was performed to provide more semiquantitative details on the Zn^2+^ solvation structure (Fig. 3f). In the positive mode ESI-MS of electrolyte **N**, the formation of Zn^2+^···TFSI^−^ complexes is indicated by a strong peak at *m*/*z* 344 corresponding to [Zn(TFSI)]^+^ and a less notable peak at *m*/*z* 408 attributable to [Zn(TFSI)]^+^ carrying two methanol molecules (the dilute), whereas only the latter type is detected for electrolytes **O** and **M**, which is reasonably ascribed to the much weaker Zn^2+^···TFSI^−^ coordination strength in these two electrolytes, as previous studies indicate [48,49]. The prevalence of [Li_3_(TFSI)_2_]^+^ (*m*/*z* 581) and [Li_4_(TFSI)_3_]^+^ (*m*/*z* 868) in electrolyte **N** is due to the elimination of H_2_O molecules in the water-dominated H_2_O···Li^+^···TFSI^−^ complexes during the ionization process. In the ESI-MS of electrolyte **M**, the strong peak at *m*/*z* 502 belonging to [(Emim)_2_TFSI]^+^ suggests the omnipresence of Emim^+^···TFSI^−^···Emim^+^ arrangements.

Theoretically, the Zn^2+^···TFSI^−^ coordination strength can be quantitatively reflected by the average intermolecular distances of TFSI^−^ anions in the primary solvation sheath of Zn^2+^, which was studied using synchrotron SAXS (schematically shown in Fig. 3g), by which more direct insight into the chemical and structural characteristics covering the occurrence and size of clusters, aggregates and networks at molecular and long-range scales can also be obtained. Concerning the liquid structure of dilute electrolytes, there is little controversy that the metal cations and anions are exclusively solvated by H_2_O molecules and are separated. As shown in Fig. 3h, the broad *I*(Q) Peak a in the low-*q* region of the SAXS profile of electrolyte **O**, centered at *q* ∼ 3.3 nm^−1^, indicates a large *d*-spacing (*d*_a_, corresponding to Peak a) between solvated TFSI^−^ of 1.9 nm (*d* = 2π/*q*) [13,21,23]. For electrolyte **N**, the formation of TFSI^−^ networks in which TFSI^−^ is directly contacted or mediated by H_2_O is indicated by the significant strengthening of Peak b, and meanwhile TFSI^−^ solvates are fully disrupted, accounting for the disappearance of Peak a. The average *d*-spacing between networked TFSI^−^ (*d*_b_) in electrolyte **N** is 0.61 nm. The corresponding value for electrolyte **M** is 0.68 nm, most likely due to the large-sized Emim^+^ ions participating in bridging the TFSI^−^ networks, as previous discussions concluded [13]. Notably, *d*_b_ here can also be the distance between neighboring Emim^+^ and TFSI^−^. Compared with Peak b, Peak c occurring in the high-*q* region is much less understood. Yu *et al.* focused on the liquid structures of concentrated LiTFSI aqueous solutions and correlated this peak to the contributions of carbon–fluorine (C–F) of TFSI^−^ anions and fluorine···fluorine (F···F) interaction between two neighboring TFSI^−^ anions [21]. We further conclude that the latter plays the dominant role in yielding Peak c, as in electrolyte **M** this peak shifts along the low-*q* direction with a sharply increased intensity much larger than Peak b. This is unlikely to be caused by the change in C–F bond length. Given that a majority of TFSI^−^ anions in electrolyte **M** reside in the primary solvation sheath of Zn^2+^, it is reasonable to conclude that intermolecular F···F interaction among Zn^2+^-coordinated TFSI^−^ anions is the key determinant of Peak c. Therefore, the low-*q* shift of Peak c from 14.5 to 13.1 nm^−1^, directing to a larger intermolecular distance (*d*_c_) of 0.43 versus 0.48 nm for electrolyte **M**, is suggestive of the weaker Zn^2+^···TFSI^−^ coordination in electrolyte **M**. We reasoned that the strong Coulombic force between Zn^2+^-coordinated TFSI^−^ and Emim^+^ might be largely responsible for such behaviors. The pronounced low-*q* peak at 5.0 nm^−1^ for electrolyte **M** corresponds to a real-space distance of ∼1.25 nm, which is unlikely to stem from specific intermolecular interactions in such a concentrated ionic system. Combining the existing extensive SAXS research results on ILs and their hybrid systems, we soundly conclude that nanoscale heterogeneity in this electrolyte due to the coexistence of ion-rich and water-rich domains accounts for this peak (termed Pre-peak or Peak p, with a characteristic distance of *d*_p_) [13,27].

The above multi-step reasoning process was distilled into a schematic workflow (Fig. S9) to enhance logical coherence and accessibility (see Note S9 for a companion summary tracing the analytical progression). Together, the above results provide sufficient information to guide us to map ‘high-resolution’ liquid pictures for the three representative electrolytes with a host of details covering solvation structure, molecular arrangement and intermolecular distance, as presented in the bottom panel of Fig. 3i.

### Liquid structure–electrochemical property correlation

We first evaluated the electrochemical stability windows of electrolytes **O, N** and **M**, with particular attention to their cathodic limits and the parasitic reactions they would experience during cathodic scan, in both two-electrode coin cells and three-electrode devices (Fig. S10). Linear sweep voltammetry (LSV) profiles recorded in the Zn||Ti cells indicate that electrolyte **M** has a significantly extended anodic limit of 2.46 V (versus Zn^2+^/Zn), 0.27 and 0.2 V above those of Zn(TFSI)_2_ saturated in water and WiS electrolyte **N**, respectively. In the cathodic scan, the rise in reduction current prior to Zn deposition occurs at 0.62 V for electrolyte **O** and 0.49 V for electrolyte **N**, combining the past studies [1,50], which are respectively attributed to HER and reduction of Zn^2+^-coordinated TFSI^−^ with minor differences in onset potential values. For electrolyte **M**, the onset of the steep cathodic current shifts to 0.11 V. Meanwhile, a intermediate Zn deposition overpotential of −0.10 V decreased by 26 mV compared to that of electrolyte **N**, demonstrating its significantly improved reduction stability and concurrent capability of facilitating Zn deposition. The remaining question is whether it follows a mechanism similar to electrolyte **N**. Previously, the research community was obliged to designate the gap between the anodic limit and the onset potential of Zn deposition as the ESW of investigated electrolytes due to the occurrence of Zn deposition often prior to HER, leading to an inaccurate evaluation of the electrochemical stability [44].

**Figure 4.**
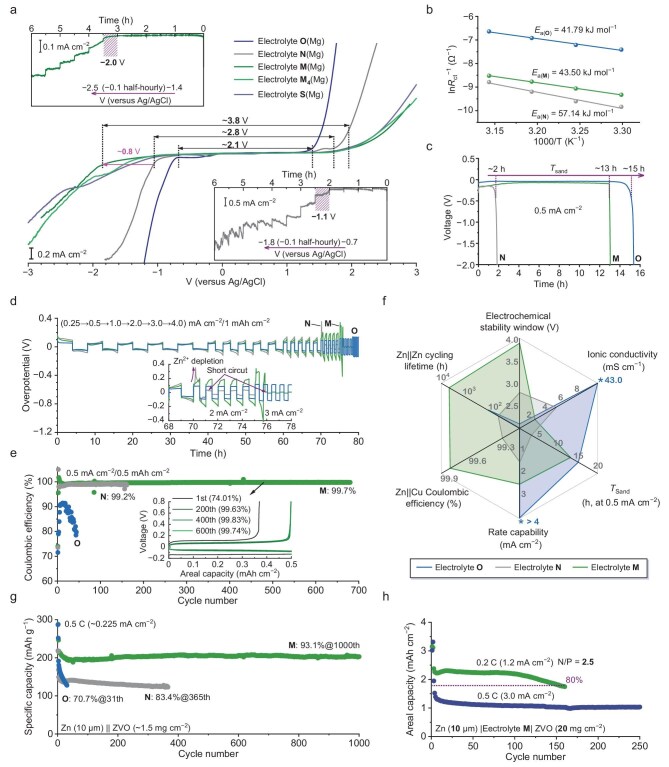
Comparative study of the electrochemical properties and Zn anode performance of different electrolytes. (a) Cathodic and anodic LSV profiles of electrolytes **O**(Mg), **N**(Mg), **M**(Mg), **M_4_**(Mg) and **S**(Mg) at 1 mV s^−1^ in a three-electrode configuration with Pt as the working and counter electrode and Ag/AgCl (in saturated KCl) as the reference electrode. Insets are the potentiostatic floating test results of electrolyte **N**(Mg) from −0.7 to −1.8 V and electrolyte **M**(Mg) from −1.4 to −2.5 V, both with an increment of −0.1 V half-hourly. (b) Arrhenius plots of *R*_ct_^−1^ at temperatures from 30°C to 45°C with 5°C increments for Zn electrode in Zn||Zn cells. (c) Galvanostatic voltage profile of Zn||Zn cells at 0.5 mA cm^−2^. (d) Rate capability of Zn||Zn cells at current densities of 0.25, 1, 2, 3 and 4 mA cm^−2^ with a fixed deposition capacity of 1 mAh cm^−2^ in each half cycle. (e) Zn plating/stripping CE in Zn||Cu cells at 0.5 mA cm^−2^/0.5 mAh cm^−2^. Inset is the plating/stripping voltage profiles of selected cycles for electrolyte M. (f) Radar plot of electrochemical properties and Zn anode cyclability afforded by investigated electrolytes. For clarity, the results exceeding the axis scale are truncated, and the exact values are given (*). (g) Cycling stability of Zn||ZVO half cells at 0.5 C rate. (h) Cycling stability of Zn||ZVO full cells at 0.2 and 0.5 C rates using electrolyte **M** (two formation cycles at 0.1 C rate).

Here, motivated by the structural and chemical similarities of Zn(TFSI)_2_ with its magnesium (Mg) analogue of Mg(TFSI)_2_, it is believed that the substitution of Zn(TFSI)_2_ with Mg(TFSI)_2_ theoretically allows for a more practical evaluation of the cathodic limits of these electrolytes due to the significantly lower reduction potential of Mg (−1.61 V versus Zn^2+^/Zn). By a combined approach of LSV (Fig. 4a) and potentiostatic floating test (insets of Fig. 4a), the cathodic limit of the electrolyte **N**(Mg) [the Mg in parentheses meaning Mg(TFSI)_2_ instead of Zn(TFSI)_2_] is determined to be in the range −1.0 to −1.1 V (versus Ag/AgCl), which for electrolyte **M**(Mg) significantly shifts to a more negative value of <−1.9 V, far below Zn deposition potential (∼−1.0 V versus Ag/AgCl). In the anodic scans, the onsets of anodic current are observable at 1.75 and 1.97 V for electrolytes **N**(Mg) and **M**(Mg), respectively. The electrochemical window of electrolyte **N**(Mg) indicated by the above results is around 2.8 V, very close to that of 21 m LiTFSI [1,51], validating the applicability of the evaluating approach we adopted. Therefore, we can reasonably conclude that electrolyte **M** has an extremely wide ESW of 3.8 V, which is among the most electrochemically stable aqueous electrolytes ever reported [47,51].

Electrolytes’ ion transport properties, electrode/electrolyte charge transfer (Zn^2+^ solvates to Zn^2+^ to Zn^0^ and vice versa) properties, and the resulting electrolyte property descriptors, including Sand’s time (*T*_sand_) and critical current density (CCD) that can heavily influence the cell performance, were characterized. Ionic conductivities (σ) of electrolytes **O, N** and **M** at 28°C were measured to be 43.0, 4.6 and 2.0 mS cm^−1^, respectively (Fig. S11). In addition to the high viscosity of the IL used, the relatively lower σ of electrolyte **M** is largely ascribed to the formation of a dense intermolecular hydrogen-bonding/electrostatic network. Of course, this is also why H_2_O molecules can be thermodynamically stabilized in such a system. The higher viscosity theoretically leads to much slower diffusion kinetics of Zn^2+^ ions in electrolyte **M**. Knowing this, it is puzzling to find that the activation energy (*E_a_*) of charge transfer resistance (*R*_ct_) for electrolyte **M** is only slightly larger than that for electrolyte **O** as it contrasts with the previously established knowledge that anionic-type solvation structures would induce higher desolvation penalties than those solvated by neutral molecules (Fig. 4b, Fig. S12) [52,53]. This leads us to further conclude that the solvation structure of Zn^2+^· ions weakly solvated by ·TFSI^−^ in electrolyte **M** has a lower desolvation energy than that of the conventional [Zn(H_2_O)_6_]^2+^. Electrolyte **N** is not competitive in either desolvation kinetics or the Zn^2+^ diffusion process, therefore showing remarkably higher *R*_ct_ and *E_a_* of *R*_ct_. *T*_sand_ of a Zn electrolyte is positively related to the concentration, diffusion coefficient and transference number of Zn^2+^, and can be indicated by the sharp electrical potential increase of a Zn||Zn symmetrical cell operating at a constant current density (above the limiting current density, or *T*_sand_ tends to infinity) [5,54]. As shown in Fig. 4c, at 0.5 mA cm^−2^, *T*_sand_ of electrolytes **O, N** and **M** is approximately estimated as 15, 2 and 13 h, respectively. Complementary to *T*_sand_, the rate capability of Zn||Zn symmetrical cells can, from another aspect, reflect the electrolytes’ Zn^2+^ ion transport ability, as seen from Fig. 4d, when the applied current densities range from 0.25–0.5 to 1.0 mA cm^−2^, an areal capacity of 1.0 mAh cm^−2^ is achievable for all three electrolytes. At 2.0 mA cm^−2^, the voltage curve for electrolyte **N** experiences a sharp increase due to Zn^2+^ depletion and then plunges because of soft shorting. Eventually, the cell fails after only one cycle. A similar result is observed for electrolyte **M** at a higher current density of 3.0 mA cm^−2^, indicating its higher critical current density of roughly 2–3 mA cm^−2^. Benefiting from the overwhelmingly high ionic conductivity, electrolyte **O** allows for the operation of Zn||Zn cells at current densities even higher than 4.0 mA cm^−2^. However, when the Zn||Zn cell is subjected to a long-term cycling test, its shortcoming of insufficient reduction stability is highlighted, and an unsatisfactory cycling stability of 158 h is delivered. The sudden overpotential increase before failure is very likely related to the accumulation of electrolytic H_2_. The more electrochemically stable electrolyte **N** is ideally free of HER, but the Zn||Zn cell using this electrolyte short-circuits earlier at ∼72 h due to dendrite issues. Electrolyte **M** can afford an ultralong lifetime of 3960 h, almost two orders of magnitude longer than those of electrolytes **O** and **N** (Fig. S13a). The overpotentials are constantly stabilized in the range of 100–200 mV, demonstrating the ability of electrolyte **M** to remain stable against reduction and facilitate uniform Zn deposition.

Zn plating/stripping reversibility in three electrolytes is quantitatively characterized in Zn||Cu asymmetrical cells, with the Coulombic efficiency (CE) versus cycle number results shown in Fig. 4e and Fig. S13b–d. The CE for electrolyte **O** can hardly achieve 90% across a short lifetime of 44 cycles, mainly due to continuous HER side reactions. For cells implementing electrolyte **N**, the cycling reversibility would not be significantly troubled by the dendrite issues before the occurrence of a short-circuit. The CE gradually reaches 99.3% and then slowly decreases to 98.7% at the end of the cycling life (162 cycles). Not surprisingly, the CE achieved with electrolyte **M** is overwhelmingly higher, averaging 99.7% (excluding the first cycle) within a significantly prolonged lifetime of over 680 cycles, which is comparable to that of the state-of-the-art electrolytes developed for Zn metal batteries (Table S3). The above results spanning various aspects of evaluating an electrolyte, from basic electrochemical properties to metal anode cyclability in terms of rate capability, reversibility and lifetime, are visualized in a radar plot shown in Fig. 4f, which hints at the huge potential of our proposed Wi(S/IL) electrolytes, represented by electrolyte **M**.

As a proof-of-concept demonstration, the practicality of electrolyte **M** was evaluated in Zn|| Zn_0.25_V_2_O_5_·nH_2_O (ZVO) cells within an operating voltage range of 0.5 − 1.4 V (versus Zn^2+^/Zn). Initially from the room-temperature galvanostatic charge/discharge test results of Zn||ZVO half cells (low cathode loading) (Fig. 4g, Fig. S13e–g), it is observed that the use of electrolyte **M** allows the Zn||ZVO cell to deliver stable discharge-specific capacities of approximately 200 mAh g^−1^ at 0.5 C and eventually a capacity retention of 93.1% over 1000 cycles (relative to the second cycle), while the cells with the other two electrolytes experience fast capacity decay or failure of overcharge in short terms. At a 1 C rate, the cell with electrolyte **M** retains 80% of its initial specific capacity (157 mAh g^−1^) over 3000 cycles and, impressively, remains operable for over 1 year (>10 000 cycles). High-rate performance is a relative shortage of electrolyte **M**, the discharge capacity at 2 C is further lowered to 120 mAh g^−1^. Other electrochemical key performance indicators afforded by electrolyte **M** such as a remarkably high capacity retention of 98.0% for fully charged Zn||ZVO cell after resting for 200 h and an ultra-wide service temperature range of −20°C to 100°C, especially a cycling durability of >700 cycles at 75°C, are hardly achievable with the existing aqueous electrolyte systems (see Fig. S14 and Note S10 for details) [55–57]. We also examined the basic electrochemical properties and battery performance of electrolytes from several different Wi(S/IL) systems (***E*3, *E*7, *E*17** and ***E*25**, indexed to Fig. S1). As detailed in Figs S15 and S16 and Note S11, the exceptionally high average Zn cycling CE of 99.85% and extremely fast charging operation of Zn batteries afforded by Zn(Emim)_2.7_(FSI)_2.7_(TFSI)_2_·4H_2_O (denoted as electrolyte **S**, ∈***E*25**) further highlights the significant generality, effectiveness and practical promise of the Wi(S/IL) strategy. Under more demanding full-cell conditions of a limited Zn source (10 μm), a high-mass-loading ZVO cathode (20 mg cm^−2^), the Zn||ZVO cell with electrolyte **M** can stably deliver an areal capacity of 2.2–2.3 mAh cm^−2^ (corresponding to a low practical negative-to positive capacity (N/P) ratio of 2.5) during the initial 100 cycles, and finally a reasonable cycle life of 160 cycles (indicated by a capacity retention of 80%) was achieved (Fig. 4h, Fig. S17).

### Chemical origins of massive improvement in Zn cyclability

**Figure 5.**
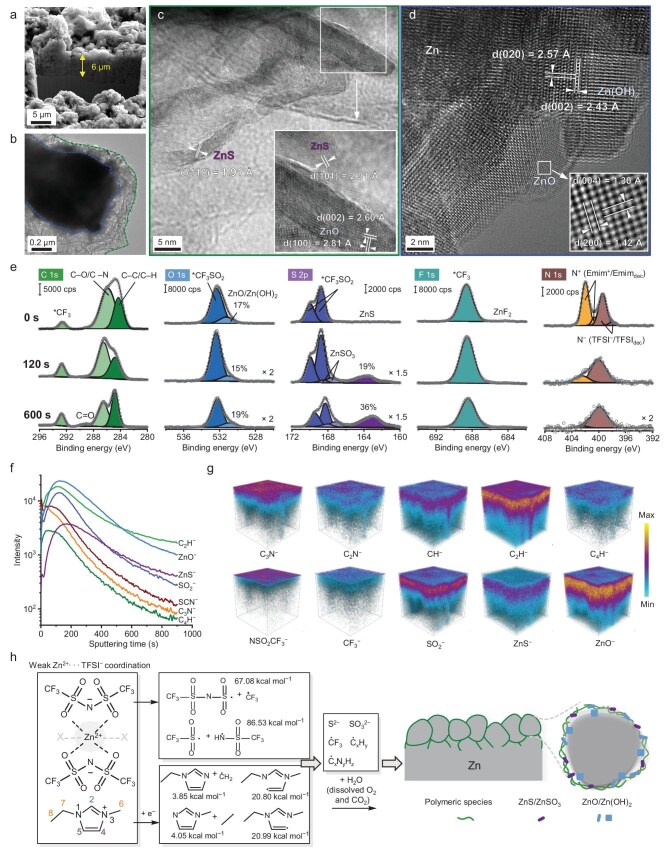
Post-mortem analysis of cycled Zn and a proposed mechanism for the SEI formation. (a) Cross-sectional SEM image of the cycled electrode etched by FIB. (b) TEM image of a single micro-sized Zn deposit, showing a dark core (blue) and semi-transparent outer layer (green). Atomic-resolution TEM images of the outer layer (c) and core (d) of Zn deposits. Insets are the zoomed-in images. (e) High-resolution C 1s, O 1s, S 2p, F 1s and N 1s depth profiling XPS spectra of the cycled electrode. (f) Depth profiles of selected fragments over sputtering time and (g) the corresponding reconstructed 3D images obtained from TOF-SIMS analysis. (h) Proposed mechanism for the reduction of Emim^+^ and TFSI^−^ weakly coordinated to Zn^2+^ to form the SEI. The formed SEI structure is schematically shown by combining all the post-mortem analysis results.

Morphological, compositional and structural analyses on the deposition layer of Zn cycled with electrolyte **M** at 1 mA cm^−2^/1 mAh cm^−2^ for 50 cycles were performed to gain a mechanistic understanding of Zn cyclability enhancement. Attributed in part to the regulating effect of non-Faradaic cation (herein Emim^+^) adsorption on the initial Zn deposition, the scanning electron microscopy (SEM) and corresponding EDS elemental mapping images of the cross-section of cycled Zn etched by a focused ion beam (FIB) indicate the microscale Zn deposits compactly stack to form the reaction layer (Fig. 5a). From the transmission electron microscopy (TEM) and EDS images of a single Zn deposit mechanically peeled from the cycled Zn, it is concluded that the Zn deposits typically feature a nano-sized electron-beam-sensitive polymeric layer (Fig. 5b). Furthermore, combining extensive analyses on the atomic-resolution TEM images of Zn deposits and the X-ray diffraction (XRD) pattern and high-resolution depth-profiling X-ray photoelectron spectroscopy (XPS) of cycled Zn, the compositional and structural features of the Zn deposits can be described as follows: the inner part is exclusively metallic Zn passivated with a ZnO-dominated surface; the outer polymeric layer is exactly composed of a TFSI anion-derived organic substrate uniformly distributed with inorganic species such as the rod-like ZnO and ZnS (Fig. 5c–e; see Figs S18 and S19 and Note S12 for additional analysis). The evolution of the N⁺ and N⁻ peaks—corresponding to contributions from Emim⁺ and TFSI^−^ and their decomposition intermediates (denoted as Emim_dec_ and TFSI_dec_)—shows a marked decrease in the N⁺ signal at greater depths, which strongly indicates the significant involvement of Emim-derived species in solid–electrolyte interphase (SEI) construction. [58,59] These XPS observations are corroborated by the time-of-flight secondary ion mass spectrometry (TOF-SIMS) analysis (Fig. 5f and g), which reveals a highly consistent picture: both Emim⁺ and TFSI^−^ undergo decomposition at the Zn surface, as indicated by the coexistence of organic fragments (e.g. C_3_N^−^, C_2_H^−^ and C_4_H^−^) and inorganic fragments (e.g. ZnS^−^ and ZnO^−^) throughout the deposition layer. The SO_2_^−^ is identified as the critical intermediate species for the formation of ZnS/ZnSO_3_. This distinctive fragment distribution confirms the formation of an organic–inorganic composite SEI. To construct a clearer mechanistic picture of how Emim⁺ and TFSI⁻ participate in SEI formation, we turn to the bond dissociation energies (BDEs) of their characteristic bonds to inform the underlying chemical mechanism. For TFSI⁻, while the C–S bond is most susceptible to cleavage, the presence of Emim⁺ significantly facilitates N–S bond scission, as suggested by the substantially lowered BDE compared to the Emim⁺-free case (Fig. S20). In contrast, the C–F bond remains robust, thereby limiting the formation of ZnF_2_, consistent with our XPS and TOF-SIMS analyses. Regarding the Emim⁺ cation, the low BDEs of representative ring bonds of N(1)–C(5) and N(3)–C(4) indicate a high propensity for ring-opening reactions to generate reactive alkene fragments (Fig. S21). This result is crucial, as it offers a direct mechanistic explanation for the prevalent presence of a polymer-rich outer SEI layer on zinc deposits. (Fig. 5h) [1,2,8,60].

Elimination of bulk-like water and formation of a weakly bonded anionic solvation sheath constitute the molecular basis for the reversible water-free Zn electrochemistry of our proposed Wi(S/IL) electrolyte; the resulting unique organic–inorganic SEI, as revealed here, physically blocks H_2_O molecules while permitting Zn^2+^ ion transport. These two mechanisms synergistically contribute to the remarkably improved Zn reversibility and durability.

## CONCLUSIONS

We experimentally show that for any popularly used Zn salts, there exist one or more ionic liquids capable of extending the salt-to-water ratio of the resulting aqueous electrolytes to the WiS regime (water-to-Zn salt ratio < 5). This strategy, termed Wi(S/IL), can significantly improve the design flexibility of WiS electrolytes by cleverly circumventing the solubility and corrosion issues associated with metal salts that provide the working ions, opening vast opportunities for developing safer and high-voltage aqueous battery systems. Among the 16 potential Wi(S/IL) systems identified from all 25 possible combinations of 5 Zn salts and 5 ILs, those featuring fluorinated sulfonyl-based anions (TFSI^−^, FSI^−^, OTf^−^) in both Zn salt and ionic liquid components tend to exhibit superior thermodynamic compatibility across a broader formulation range, which facilitates the compositional tunability and microscopic structure of the Wi(S/IL) electrolytes and the achievement of desirable electrochemical properties. As a proof-of-concept demonstration, the Zn(TFSI)_2_–EmimTFSI–H_2_O system was chosen for the comprehensive structure–property–performance relationship study due to its large single-phase liquid region and minimal ionic components (containing only one anionic species). By combining IR/NMR/Raman spectroscopy, EIS-MS and synchrotron SAXS, it was confirmed that, in dilute aqueous electrolytes of Zn(TFSI)_2_ (represented by electrolyte **O**), Zn^2+^ ions are typically solvated by six H_2_O molecules; with the heavy addition of LiTFSI to form electrolyte **N**, the primary solvation sheath of Zn^2+^ ions becomes TFSI-dominated and meanwhile that of Li^+^ ions remains a water-dominated state; for the Wi(S/IL) electrolyte system of Zn(TFSI)_2_–EmimTFSI–H_2_O (electrolyte **M** being the optimal formulation), Zn^2+^ ions preferentially coordinate with TFSI^−^ regardless of the electrolyte formulations, conclusively demonstrating that Zn^2+^ ions featuring a high charge-to-size ratio have a strong propensity to form a TFSI-dominated primary solvation sheath, which contrasts sharply with the situation in Li-based WiS or Wi(S/IL) electrolytes where H_2_O molecules always preferentially occupy the first solvation sheath of Li^+^ ions. All these experimental findings show excellent consistency with our MD simulation results. The unique anionic solvation structure of Zn^2+^ ions and the resulting anion-derived, protective organic–inorganic SEI layer are identified as the two key factors enabling exceptional ESW of 3.8 V and Zn plating/stripping reversibility of 99.7%. To conclude, we demonstrate the superior design flexibility of Wi(S/IL) electrolytes and develop a combined experimental approach effective in depicting their liquid structures. By elucidating how component nanostructuring governs electrolyte properties, optimal electrochemical performance through the Wi(S/IL) approach becomes predictably achievable. We thus envision that this systematic study focusing on Wi(S/IL) electrolytes will revolutionize the research of aqueous electrolytes and beyond.

## Supplementary Material

nwag125_Supplemental_File
