# Supplementary material for: Experimentally tracing water molecules and mapping high-resolution liquid structure pictures for promising water-in-salt/ionic liquid electrolytes
Source: Natl Sci Rev. 2026 Mar 6;13(8):nwag125. doi: 10.1093/nsr/nwag125 (PMC13131236; doi:10.1093/nsr/nwag125)
Supplement: nwag125_Supplemental_File [file nwag125_supplemental_file.pdf]

## Supplementing Information for

# Experimentally tracing water molecules and mapping high-resolution liquid structure pictures for promising water-in-salt/ionic liquid electrolytes

Le Yu[1,†,*], Sijun Wang[1,†], Jing Huang[1], Xuanyu Zeng[1], Xiaoyan Zhou[1,2], Zhiqiang Wang[1], Junqing Chen[1], Jiliang Liu[3] and Chaoji Chen[1,*]

[1]School of Resource and Environmental Sciences, Hubei Biomass-Resource Chemistry and Environmental Biotechnology Key Laboratory, Wuhan University, Wuhan 430079, China;

[2]School of Science, Hubei University of Technology, Wuhan 430070, China;

[3]European Synchrotron Radiation Facility (ESRF), Grenoble 38000, France

***Corresponding authors.** E-mails: yuleee@whu.edu.cn; chenchaojili@whu.edu.cn

[†]Equally contributed to this work.

**Keywords**: water-in-salt electrolytes, liquid structure, ionic liquids, solvation sheath, electrochemistry

## METHODS

### Electrolyte characterization

Fourier transform infrared (FTIR) spectra were recorded with a Bruker Vertex 70 Spectrometer with the platinum Attenuated Total Reflection (ATR) accessory (diamond crystal). $^{1}$H NMR measurements were performed using a 600 MHz liquid NMR spectrometer (Avance Neo 600, Bruker Corporation), equipped with a coaxial NMR tube (Wilmad-LabGlass) containing a 10:90 vol/vol mixture of 2,2,3,3-tetrafluoropropanol with (methyl sulfoxide)-d6 as an internal reference. Raman spectra were recorded with a laser excitation wavelength of 638 nm. (XploRa PLUS, HORIBA Jobin Yvon). All of the above measurements were carried out with the temperature controlled at 30 ± 1°C. Electrospray ionization mass spectrometry (ESI-MS) spectra were acquired with an Orbitrap Elite LTQ XL (Thermo Fisher Scientific), operating in the positive (or negative) ion mode, equipped with a Z-spray source. All analytes were further diluted 10000 × in methanol prior to analysis. SAXS measurements were carried out at DUBBLE beamline BM26 at 12 keV (wavelength 1.033Å). The beam size 400 × 600 μm focused at the detector position with $5 \times 10^{10}$ photons $s^{-1}$. The sample to detector distance was set to 466.9 mm. 2D SAXS patterns were recorded using a Pilatus Detector. The data reductions were calculated by customized software.

### Electrochemical measurements

$Zn_{0.25}V_2O_5 \cdot nH_2O$ (ZVO) was synthesized by a hydrothermal method [1]. Poly(3,4-ethylenedioxythiophene)-coated $V_2O_5$ ($V_2O_5$@PEDOT) was prepared via a mild solution synthesis process [2]. $V_2O_5$@PEDOT and ZVO cathodes with various active material mass loadings (1.5–20 mg $cm^{-2}$) for half-cell and full-cell tests were all prepared via a vacuum filtration method. 10 wt% bacterial cellulose and 10 wt% multiwalled carbon nanotubes (purchased from Guangdong Canrd New Energy Technology Co., Ltd.) were incorporated to ensure structural integrity and electrical conductivity. All two-electrode configurations, including Zn||Zn, Zn||Cu, Zn||ZVO, and Zn||$V_2O_5$@PEDOT for the cyclability and/or electrochemical impedance spectroscopy (EIS) tests, Zn||Ti for the linear sweep

voltammetry (LSV) tests, were assembled in CR2032-type coin cells, using a pair of Whatman GF/A glass fiber membranes as the separator. LSV and potentiostatic floating tests in a three-electrode configuration (a Pt foil as working, a separate Pt foil as counter electrodes, and an Ag/AgCl (in saturated KCl aqueous solution) as reference electrode) were performed on a CHI760E electrochemical workstation (CH Instruments). For post-mortem analysis, the Zn||Zn cell was assembled in a quartz glass cuvette with an inter-electrode distance of ca. 1.5 mm without the use of a separator. All galvanostatic cycling tests were conducted with LAND CT2003A. Zn foils (10 and 100 μm) were polished using 2000 grit sandpapers and washed with deionized water before cell assembly. The environmental temperature for all the cycling tests is 30°C if not specified.

**Post-mortem analysis of Zn**

Back scattered electron (BSE) images and wavelength dispersive spectroscopy (WDS) elemental maps were acquired on a JXA-8530F Plus Field Emission Electron Probe Micro-analyzer (JEOL). The cross-sectional scanning electron microscope (SEM) image of the Zn deposition layer was observed via a focused ion beam system (XEIA3 GMU SEM/Plasma-FIB, Tescan). Powder X-ray diffraction (XRD) tests were conducted on a Rigaku SmartLab SE diffractometer with Cu Kα radiation. The chemical change of the cycled Zn surface was analyzed using X-ray photoelectron spectroscopy (XPS, ESCALAB 250Xi, Thermo Scientific) and time-of-flight secondary ion mass-spectroscopy (TOF-SIMS, IONTOF M6 instrument) with a $Bi_3^{2+}$ gun (30 keV, 0.43 pA). The C1s peak (284.8 eV) was used as the reference to calibrate other binding energy values. The contents of different species in the SEI layers were obtained by fitting the XPS spectra using Avantage 5.976 software. The etching rate for the Ar sputtering is estimated to be ≈ 0.4 nm $s^{-1}$. Transmission electron microscopy (TEM) imaging and energy-dispersive X-ray spectroscopy (EDX) mapping of the Zn deposits were acquired on a JEOL JEM-ARM200F microscope operated at 200 kV.

**Molecular dynamics (MD) simulations**

MD simulations were carried out using GROMACS 2018. 4 package. The solvation structure of $Zn^{2+}$ and $Li^{+}$ in 1 m $Zn(TFSI)_2$ and 1 m $Zn(TFSI)_2$ + 20 m LiTFSI electrolyte was investigated with the general AMBER force field (GAFF) [3, 4]. The MD parameters for $Zn^{2+}$ and $Li^{+}$ were in the Merz force-field parameters [5]. The solvation structure of $Zn^{2+}$ in an ionic-liquid-containing electrolyte was investigated with Optimized Potentials for Liquid Simulations force field (OPLS-AA/M), and the MD parameters for $Zn^{2+}$ were in the built-in parameters. Water molecules in all electrolyte systems were simulated in the TIP4P model [6]. The MD parameters for $EMIM^{+}$ and $TFSI^{-}$ were acquired from the optimized potentials for liquid simulation-ionic-liquid virtual site forcefield (OPLS-VSIL), developed by Orlando Acevedo (https://github.com/orlandoacevedo/IL/) [7]. The corresponding atom charges of each component were based on 0.8-scaling charges [8]. The constant-pressure and temperature (NPT) ensembles were first performed at 300 K for 20 ns to ensure system equilibrium, and then another NPT run of 5 ns was used for the post-analysis. The radial distribution functions (RDF) of $Zn^{2+}$ and $Li^{+}$ cations were calculated from the built-in module in the GROMACS package. The MD simulation visualization was achieved by visual molecular dynamics (VMD) [9]. The parameters of the simulated electrolyte are listed in Table S4.

**Bond dissociation energy (BDE) calculations**

BDE calculation is one of the most direct methods for quantifying the strength of chemical bonds, defined as the energy required to homolytically cleave a chemical bond into free radicals [10]. The BDE of the chemical bond in $TFSI^{-}$ and $Emim^{+}$ was calculated through the Gaussian 09 package. All structures of molecules were optimized with B3LYP/6-311++G (d, p) for C, O, N, H, F, and S elements, and B3LYP/SDD for the Zn element. Vibrational frequency calculations at the same level of theory were performed on all optimized structures to confirm stationary points as minima [11]. Take the homolytic cleavage of molecule AB to generate two free radical fragments A· and B· (A–B → A·+B·) as an example, the BDE between two components (A·, B·) is defined as the enthalpy change during the process of chemical bond cleavage:

$$BDE = H_{A\bullet} + H_{B\bullet} - H_{A-B}$$

Where $H_{(A-B)}$ represents the total enthalpy of the parent molecule, and $H_{(A\cdot)}$ and $H_{(B\cdot)}$ are the enthalpy values of the two radical fragments after the bond cleavage. When the bonds of molecules break but do not separate into two free radicals, the dissociation energy of the bond is counted as the change in enthalpy before and after the bond breakage. The resulting products from bond cleavage were fully optimized to their minimum energy states. The final image of the simulation species was processed by Vesta software [12].

## SUPPLEMENTARY NOTES

1. Details for the preparation of various Wi(S/IL) electrolyte systems. ............................................S7

2. Determination of the ternary solubility diagram of $Zn(TFSI)_2$-EmimTFSI-$H_2O$. ..........................S9

3. General considerations about the selection of electrolyte samples from the liquid region...........S10

4. Interpretations of FTIR spectra in the 1400–1800 $cm^{-1}$ region. .................................................S12

5. Quantifying the contributions of the four component modes in the O–D stretching region. ........S15

6. More detailed discussion on the liquid structure evolution of the $Zn(TFSI)_2$-EmimTFSI-$H_2O$ electrolytes with formulations on lines **MC** and **MB**. ..................................................................S17

7. Comparison of MD-simulated and FTIR-derived numbers of $H_2O$ in the primary solvation sheath of $Zn^{2+}$ as a function of electrolyte formulation. ...................................................................S20

8. Correlating water $^1H$ chemical shift to Hofmeister effect in various Zn electrolytes. ..................S23

9. Multispectroscopic analysis summary for elucidating the liquid structures. ................................S25

10. Studies on long-term cyclability, rate capability, storage performance, and operability at extreme temperature conditions. ...........................................................................................................S31

11. Rate and cycling performance of a Wi(S/IL) eletrolyte belonging to the $Zn(TFSI)_2$-EmimFSI-$H_2O$ (***E*25**) system in Zn||$V_2O_5$@PEDOT cells. .........................................................................S33

12. Additional discussions on Zn cycling interphasial chemistry. ...................................................S38

References .........................................................................................................................S47

**Note S1. Details for the preparation of various Wi(S/IL) electrolyte systems.**

Zn salts: zinc acetate ($Zn(Ac)_2$, Macklin), zinc trifluoromethanesulfonate ($Zn(OTf)_2$, Aladdin), zinc sulfate heptahydrate ($ZnSO_4{\cdot}7H_2O$, Aladdin), zinc chloride ($ZnCl_2$, Aladdin), and zinc bis(trifluoromethanesulfonyl)imide ($Zn(TFSI)_2$, TCI); ILs: 1-ethyl-3-methylimidazolium acetate (EmimAc), 1-ethyl-3-methylimidazolium trifluoromethanesulfonate (EmimOTf), 1-ethyl-3-methylimidazolium dicyanamide (EmimDCA), 1-ethyl-3-methylimidazolium dicyanamide (EmimDCA), 1-ethyl-3-methylimidazolium bis(trifluoromethanesulfonyl)imide (EmimTFSI, Lanzhou Greenchem ILs), 1-ethyl-3-methylimidazolium bis(fluorosulfonyl)amide (EmimFSI, Lanzhou Greenchem ILs). All these chemicals were used as received.

These five zinc salts and five ILs were combined in a full-factorial design, yielding 25 distinct Wi(S/IL) electrolyte systems. These are arranged in a 5×5 matrix where each row corresponds to a specific zinc salt and each column to a specific ionic liquid. The systems are sequentially numbered from ***E*1** to ***E*25** in a row-wise manner (left to right across each row). In a typical electrolyte preparation procedure, 0.1 g Zn salt was added to 1 g IL to evaluate the solubility. As shown in Figure S1, all five Zn salts are quite soluble (far exceeding 0.1 g/1 g) in the IL of EmimAc, and the addition of water would not cause phase separation, which is critical for the obtainment of Wi(S/IL) electrolytes with a desirable low water-to-salt ratio. Next is EmimDCA, which can form Wi(S/IL) electrolyte systems with four Zn salts. From the angle of Zn salt, $Zn(TFSI)_2$ is easily soluble in all the ILs examined, and similarly, all the $Zn(TFSI)_2$/IL mixtures can form homogeneous Wi(S/IL) electrolytes. Added up, without water, fourteen among twenty-five combinations can be homogeneous solutions (case (1)). The special cases are the combinations of $Zn(OTf)_2$ with EmimTFSI or EmimFSI, which become miscible with the introduction of water. These two are categorized as case (2) of Wi(S/IL) electrolytes and have proven to be promising electrolyte candidates in our previous work. Although all these sixteen systems can reach the WiS regime (water-to-salt ratio < 5), some of them might be excluded for failing to fulfill the general requirements of Zn electrolytes, such as the compatibility with battery component (namely

corrosiveness) and the concentration of $Zn^{2+}$ (>~1 M is preferred).

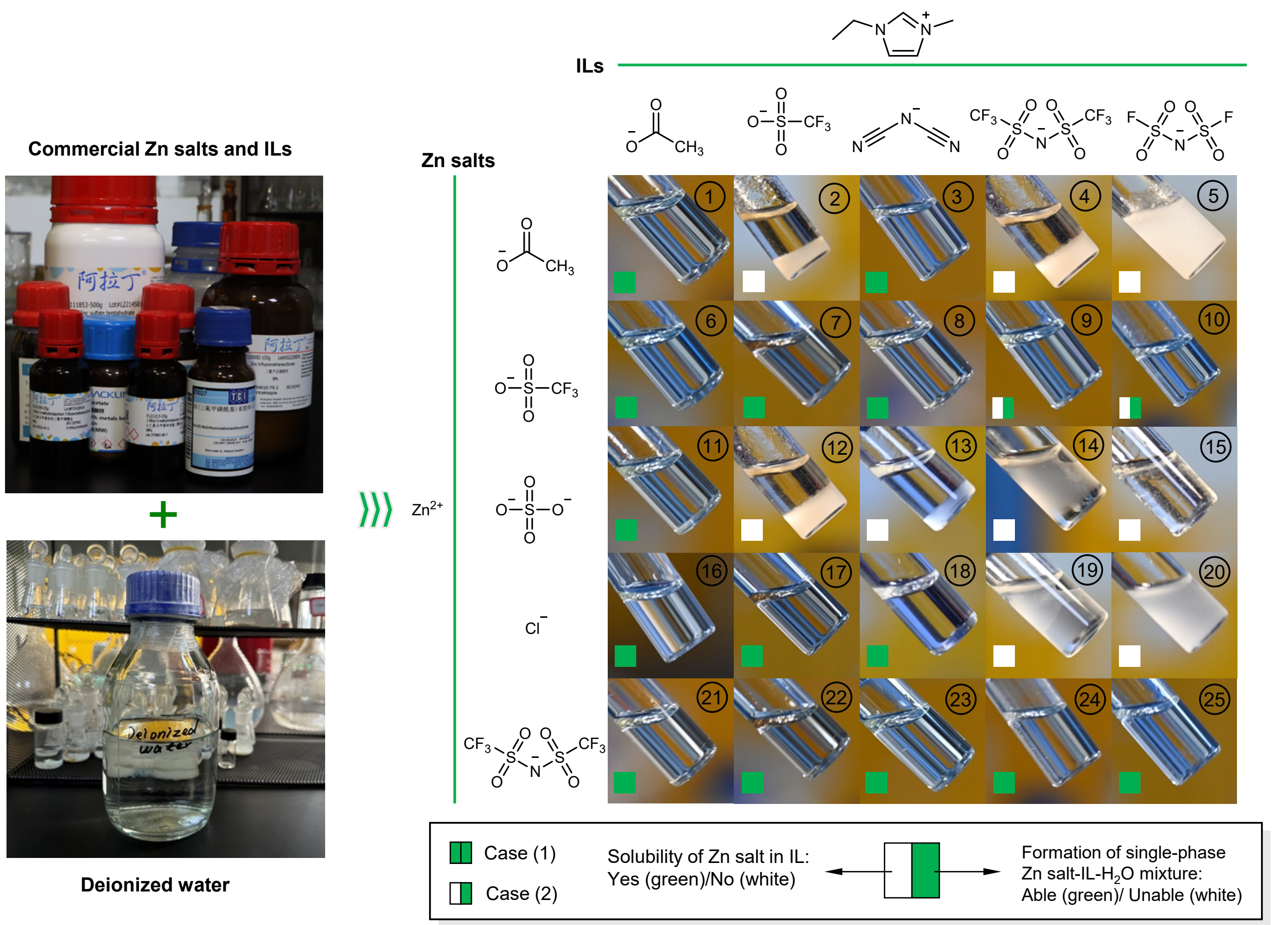


**Figure S1**. Digital photographs showing the appearances of twenty-five Zn salt-IL-$H_2O$ mixtures prepared by freely combining five popularly used Zn salts and five commercial ILs. Sixteen single-phase liquid Wi(S/IL) electrolyte systems could be obtained. Based on the characteristic solubility behaviors, two of them — $Zn(OTf)_2$-EmimTFSI-$H_2O$ (***E*9**) and $Zn(OTf)_2$-EmimFSI-$H_2O$ (***E*10**)— are examples of case (2), and the remaining fourteen fall into case (1). Anions of Zn salts $Ac^-$, $OTf^-$, $SO_4^{2-}$, $Cl^-$, and $TFSI^-$ from top to bottom; Anions of Emim-based ILs are $Ac^-$, $OTf^-$, $DCA^-$, $TFSI^-$, and $FSI^-$ from left to right.

**Note S2. Determination of the ternary solubility diagram of $Zn(TFSI)_2$-EmimTFSI-$H_2O$.**

As shown in Figure 2a, the dashed “solubility line” of this TFSI-based Wi(S/IL) electrolyte system was obtained by connecting the points of saturated $Zn(TFSI)_2$ in $H_2O$ with a solubility of 4.4 mol $kg^{-1}$ (labeled as **D** ) and in EmimTFSI with a solubility of 0.89 mol $kg^{-1}$ (labeled as **E**), indicating the ideal solubility limit for the ternary mixtures. Practically, in tens of trials, electrolytes with formulations located in this quadrilateral region (encompassing those on the “solubility line” **DE**) are exclusively shown to be single-phase liquid. On the other side, we failed to prepare a single-phase liquid electrolyte with formulation beyond the quadrilateral region due to the hydrotropic effect, a behavior totally unlike that of the LiTFSI-EmimTFSI-$H_2O$ electrolyte system. Hence, the quadrilateral **BCDE** (highlighted in light cyan, **B** and **C** represent pure components of EmimTFSI and $H_2O$, respectively) was approximatively determined to be the practical liquid region (the remaining is referred to as the phase segregation region). The absence of hydrotropic effect (excess solubility) ever reported for the LiTFSI-EmimTFSI-$H_2O$ electrolyte system suggests that a minor change in components of such ternary systems can substantially influence the solubility behavior and further the electrochemical performance.[13] Hence, a “zero-based” investigation is warranted for deciphering the liquid structure of the $Zn(TFSI)_2$-EmimTFSI-$H_2O$ electrolyte system (the general formula is $Zn(Emim)_x(TFSI)_{x+2}\cdot nH_2O$).

**Note S3. General considerations about the selection of electrolyte samples from the liquid region.** Before the experimental investigation, it is essential to screen out an electrolyte formulation that can show the optimal or near-optimal overall electrochemical properties. This is possible by leveraging the basic rules generalizable to ternary phase diagrams and the existing vast knowledge of WiS electrolyte chemistries. First, compared to the inner liquid region, on the "solubility line" **DE**, we are more likely to find an electrolyte formulation that can deliver the optimal electrochemical stability. Additionally, the electrolyte formulation should be neither too close to **D** (liable to HER) nor to **E** (low Zn concentration and low ionic conductivity). Combining the above two points, it was found mathematically that both $x$ and $n$ in the formula $Zn(Emim)_x(TFSI)_{x+2}\cdot nH_2O$ of the electrolyte prepared with **D** and **E** in a mass ratio of 5.7: 1 happen to be almost integers, specifically, $x = 2$ and $n = 4$ (strictly, 3.9). Such an integral stoichiometric ratio is of practical significance. Starting from this formulation ($Zn(Emim)_2(TFSI)_4\cdot 4H_2O$, labeled as **M** in Figure 2a), we can easily create a control group of electrolytes also having integral stoichiometric ratios along a certain line in the ternary phase diagram: formulations on line **MC** are characterized by a constant $x$ value of 2 (e.g., $\mathbf{M_1}$: $Zn(Emim)_2(TFSI)_4\cdot 6H_2O$; $\mathbf{M_2}$: $Zn(Emim)_2(TFSI)_4\cdot 8H_2O$; $\mathbf{M_3}$: $Zn(Emim)_2(TFSI)_4\cdot 10H_2O$); $n$ values of formulations on line **MB** are invariably 4 (e.g., $\mathbf{M_4}$: $Zn(Emim)_4(TFSI)_6\cdot 4H_2O$; $\mathbf{M_5}$: $Zn(Emim)_8(TFSI)_{10}\cdot 4H_2O$; $\mathbf{M_6}$: $Zn(Emim)_{16}(TFSI)_{18}\cdot 4H_2O$; $\mathbf{M_7}$: $Zn(Emim)_{32}(TFSI)_{34}\cdot 4H_2O$).

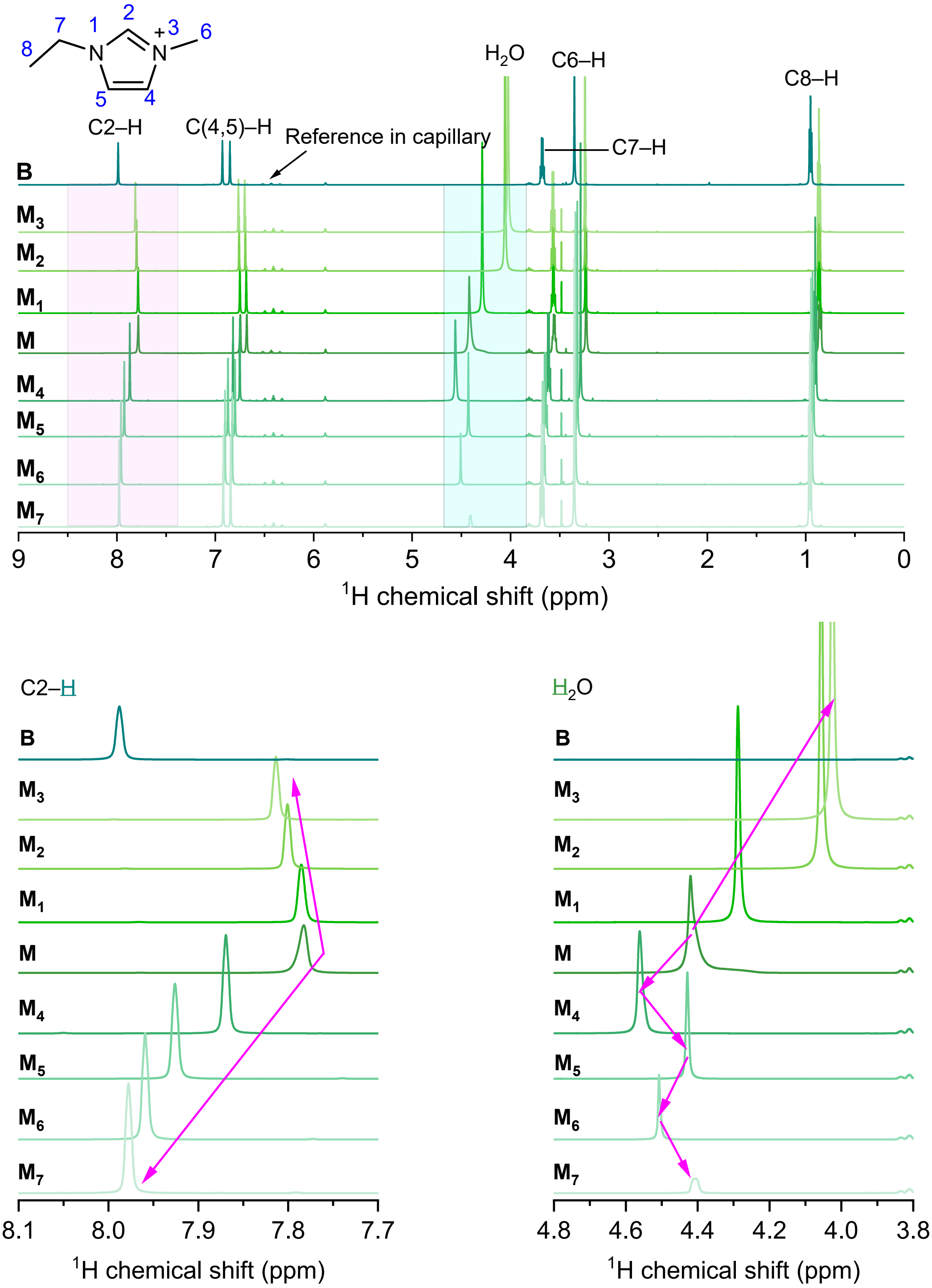


**Figure S2**. $^{1}H$ NMR spectra of EmimTFSI, $Zn(Emim)_2(TFSI)_4 \cdot nH_2O$ ($n$ = 4, 6, 8, and 10, respectively correspond to **M**, **$M_1$**, **$M_2$**, and **$M_3$**), and $Zn(Emim)_x(TFSI)_{x+2} \cdot 4H_2O$ ($x$ = 4, 8, 16, and 32, respectively correspond to **$M_4$**, **$M_5$**, **$M_6$**, and **$M_7$**). The bottom panels are zoomed-in spectra of C2–<u>H</u> and <u>H</u>$_2$O. Reference in capillary is a 10:90 vol/vol mixture of 2,2,3,3-tetrafluoropropanol with (methyl sulfoxide)-d6.

**Note S4. Interpretations of FTIR spectra in the 1400–1800 $cm^{-1}$ region.**

Unlike the O–H stretching mode, it is found that the O–H bending vibration of water molecules (at ~1630 $cm^{-1}$) is not sensitive to the change in the surrounding environment. Slight shifts from 1628 to 1631 $cm^{-1}$ are observed as the *n* value for $Zn(Emim)_2(TFSI)_4 \cdot nH_2O$ decreases from 10 to 4 (Figure S3a). The band centered at 1573 $cm^{-1}$ is assigned to the C=N stretching vibration in the imidazolium ring[14, 15]; bands at 1472, 1457, and 1432 $cm^{-1}$ are assigned to the C–H bending vibration of the imidazolium cation[16]. The positions of all these characteristic bands belonging to the imidazolium cation remain invariable in electrolyte **M**→$\mathbf{M_7}$ (Figure S3a–c). The O–H bending vibration band of $Zn(Emim)_2(TFSI)_4 \cdot nD_2O$ strengthens with decreasing *n* value, showing the same trend as the stretching mode. This again consolidates that water molecules strongly interact with the imidazolium cation (mainly C2–H) instead of coordinating with $Zn^{2+}$. The O–H bending vibration for electrolytes **O**, **D**, **M**, and **N** all shift along the low-frequency direction to different extents relative to that of pure water, showing no obvious dependence on electrolyte composition or salt concentration, which provides limited information for further analysis (Figure S3d). The reason for the occurrence of O–H species (3000–3800 and 1400–1550 $cm^{-1}$) in the spectra of electrolytes **O'** and **N'** remains unclear (Figure S3e).

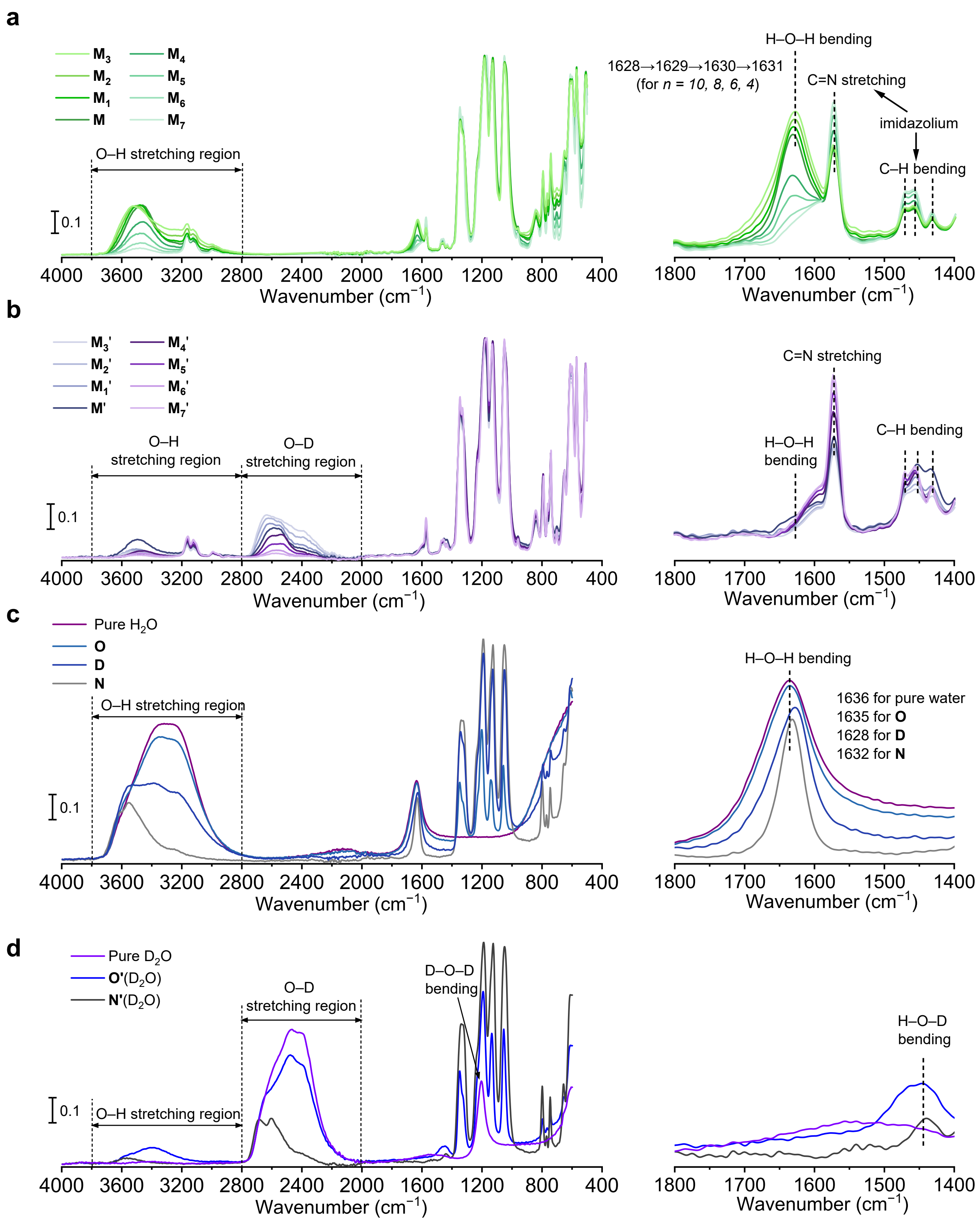


**Figure S3. FTIR spectra of different electrolytes recorded between 400 and 4000 cm$^{-1}$.** (a) $Zn(Emim)_2(TFSI)_4 \cdot nH_2O$ ($n$ = 4, 6, 8, and 10, respectively corresponding to electrolytes **M**, **M$_1$**, **M$_2$**, and **M$_3$**) and $Zn(Emim)_x(TFSI)_{x+2} \cdot 4H_2O$ ($x$ = 4, 8, 16, and 32, respectively corresponding to

electrolytes $\mathbf{M_4}$, $\mathbf{M_5}$, $\mathbf{M_6}$, and $\mathbf{M_7}$). (b) $Zn(Emim)_2(TFSI)_4 \cdot nD_2O$ ($n$ = 4, 6, 8, and 10, respectively corresponding to electrolytes **M'**, $\mathbf{M_1'}$, $\mathbf{M_2'}$, and $\mathbf{M_3'}$) and $Zn(Emim)_x(TFSI)_{x+2} \cdot 4D_2O$ ($x$ = 4, 8, 16, and 32, respectively corresponding to electrolytes $\mathbf{M_4'}$, $\mathbf{M_5'}$, $\mathbf{M_6'}$, and $\mathbf{M_7'}$). (c) Pure $H_2O$, electrolyte **O** (1 m $Zn(TFSI)_2$ in $H_2O$), electrolyte **D** (4.4 m $Zn(TFSI)_2$ in $H_2O$), and electrolyte **N** (1 m $Zn(TFSI)_2$ + 20 m LiTFSI in $H_2O$). (d) Pure $D_2O$, electrolyte **O'** (1 m $Zn(TFSI)_2$ in $D_2O$), and electrolyte **N'** (1 m $Zn(TFSI)_2$ + 20 m LiTFSI in $D_2O$). The right panels are the corresponding zoomed-in spectra between 1400 and 1800 $cm^{-1}$.

**Note S5. Quantifying the contributions of the four component modes in the O–D stretching region.**

It is serendipitously found that, in addition to the occurrence of band shift to lower wavenumbers with variations at each step ($n$ =10 → 8 → 6 → 4) almost identical to those of the $Zn(Emim)_2(TFSI)_4 \cdot nH_2O$ electrolytes, the intensity of water O–H stretching band increases continuously with the decreasing $n$ value, particularly notable from 6 to 4, indicating the formation of higher abundance of HOD through intense H/D exchange between C2–H and $D_2O$ at lower water concentrations. This is one more solid piece of evidence that, with decreasing water concentration, water molecules have a greater tendency to disengage from the primary $Zn^{2+}$ solvation sheath and interact with $Emim^+$ for sure, $TFSI^-$, and other water molecules via hydrogen bonding. Despite the H/D exchange reaction, water in $Zn(Emim)_2(TFSI)_4 \cdot nD_2O$ (labeled as **M'**, **$M_1$'**, **$M_2$'**, and **$M_3$'** for n = 4, 6, 8, and 10, respectively) consists of mainly $D_2O$ due to the overwhelmingly high D-atom to C2–H ratio. Hence, in the water O–D stretching region of 2000–2800 $cm^{-1}$ (overall shifted by approximately 1000 $cm^{-1}$ relative to the O–H stretching band), very similar spectra evolution as a function of $n$ value is observed. Minimal disparities in relative positions and contributions of the sub-bands can be mainly attributed to the different physical natures between $H_2O$ and $D_2O$. Overall, as the spectrum deconvolution results of pure $D_2O$ indicate, the water O–D stretching bands can be deconvoluted into four sub-bands at ca. 2391, 2523, 2616, and 2681 $cm^{-1}$ and interpreted in the same way as that of the water O–H stretching band, namely $\nu_s$OD, $\nu_{as}$OD, $\nu OD^b$, and $\nu OD^f$ from lower to higher wavenumbers. Based on this, the contribution of each O–D stretching mode of $Zn(Emim)_2(TFSI)_4 \cdot nD_2O$ electrolytes is quantitatively determined and visualized in the bar chart (right top of **Figure 2d**).

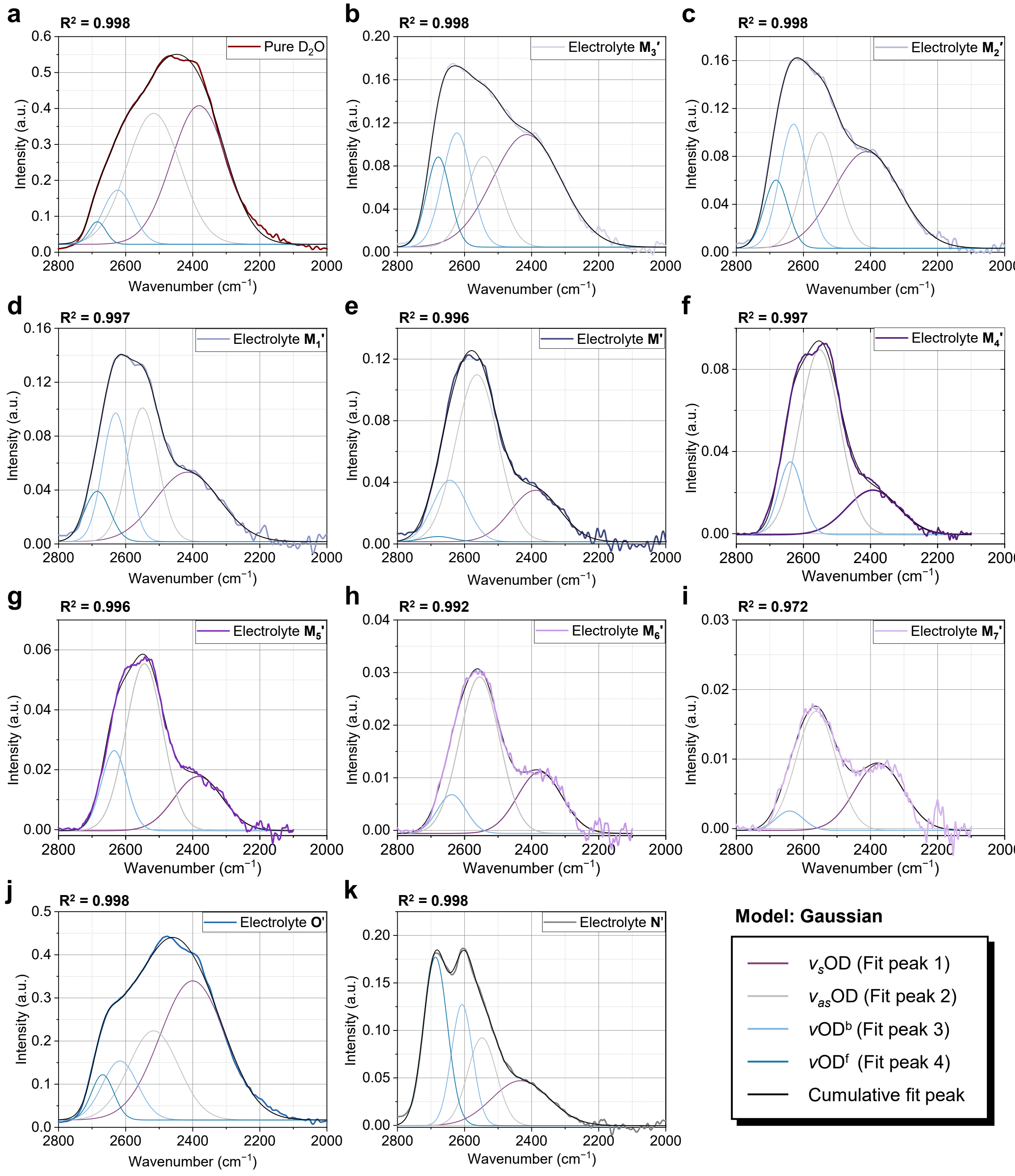


**Figure S4**. Deconvolution of O–D stretching band of (a) Pure $D_2O$ and electrolytes (b) **$M_3$'**, (c) **$M_2$'**, (d) **$M_1$'**, (e) **M'**, (f) **$M_4$'**, (g) **$M_5$'**, (h) **$M_6$'**, (i) **$M_7$'**, (j) **O'**, (k) **N'** using Gaussian function.

**Note S6. More detailed discussion on the liquid structure evolution of the $Zn(TFSI)_2$-EmimTFSI-$H_2O$ electrolytes with formulations on lines MC and MB.**

Initially, for $n$ = 10 (electrolyte $\mathbf{M_3}$ on line **MC**), the number of water molecules in the primary $Zn^{2+}$ solvation sheath is 3.4, estimated by multiplying $n$ with the combined area percentage of $\nu OD^b$ and $\nu OD^f$ (these two items together are equivalent to $H^f$-D–$H^b$), indicating a water-dominated solvation sheath with, possibly, one $TFSI^-$ anion, somewhat like the dilute aqueous electrolytes; the area percentage of $\nu OD^f$ is lowered while that of $\nu OD^b$ increases for the medium water concentration cases (electrolyte $\mathbf{M_2}$ and $\mathbf{M_1}$). Higher $\nu OD^b$ is a signal that $Zn^{2+}$ coordinated water molecules are increasingly being hydrogen-bonded with molecular species ($H_2O$ and $TFSI^-$) outside the solvation sheath, meanwhile $TFSI^-$ progressively competes with water in coordinating $Zn^{2+}$, forming typical Zn coordinated $H_2O/TFSI^-$ complexes; lastly, the number of $Zn^{2+}$ coordinated water molecules drastically decreases to as low as 0.76 when $n$ = 4 (electrolyte **M**), more strikingly is that $\nu OD^f$ almost disappears in this case, which further suggests the few water molecules in the primary solvation sheath is only very weakly coordinated with $Zn^{2+}$. In other words, water molecules are largely excluded, and the $Zn^{2+}$ solvation sheath becomes $TFSI^-$-dominated. The notably high area percentage of $\nu_{as}OD$ (ca. 59%) for $n$ = 4 can be rationalized by an assumption that water molecules excluded from the solvation sheath are apt to be hydrogen-bonded with one or two $TFSI^-$ anions and one $Emim^+$ cation by donating two hydrogen bond donors and one hydrogen bond acceptor (an example of such bonding motifs is highlighted using a grey cycle in **Figure 2e**).

An enlarged view of O–H stretching vibration for electrolytes $\mathbf{M}\rightarrow\mathbf{M_7}$ is shown in Figure S5a (upper panel), compared to electrolytes $\mathbf{M_3}\rightarrow\mathbf{M}$ with formulations on line **MC**, the peak position of O–H stretching vibration band of the electrolytes $\mathbf{M}\rightarrow\mathbf{M_4}\rightarrow\mathbf{M_7}$ marginally shifts. To corroborate this, FTIR spectra of electrolytes $\mathbf{M_4'}\rightarrow\mathbf{M_7'}$ (the $D_2O$ counterparts of electrolytes $\mathbf{M_4}\rightarrow\mathbf{M_7}$) were obtained for quantifying the contribution of sub-bands. Firstly, a collective presentation of FTIR spectra of electrolytes $\mathbf{M'}\rightarrow\mathbf{M_7'}$ was generated for visual comparison (bottom panel of Figure S5a). As seen, the

overall peak shift trend agrees well with that of the electrolytes **M**→$\mathbf{M_7}$: a continuous redshift is observed for electrolytes $\mathbf{M_3'}$→**M'**, and then the peak intensity decays monotonically as the water concentration is diluted by the addition of EmimTFSI ($x = 2 \rightarrow 32$). For further clarification, the O–D stretching vibration band of electrolytes $\mathbf{M_4'}$→$\mathbf{M_7'}$ was deconvoluted using Gaussian function (Figure S5b). Among the three sub-bands, $\nu_{as}$OD is invariably the dominant species. The weak sub-band of $\nu OD^b$ becomes almost unobservable for $\mathbf{M_7'}$, indicating the improbability of water molecules coordinating with $Zn^{2+}$ ions. The area percentage of $\nu_s$OD, representing the abundance of bulk-like water, continuously increases from 20% for $\mathbf{M_4'}$ to 38% for $\mathbf{M_7'}$, suggesting the overall tendency of clustering of water molecules residing outside the primary solvation sheath of $Zn^{2+}$.

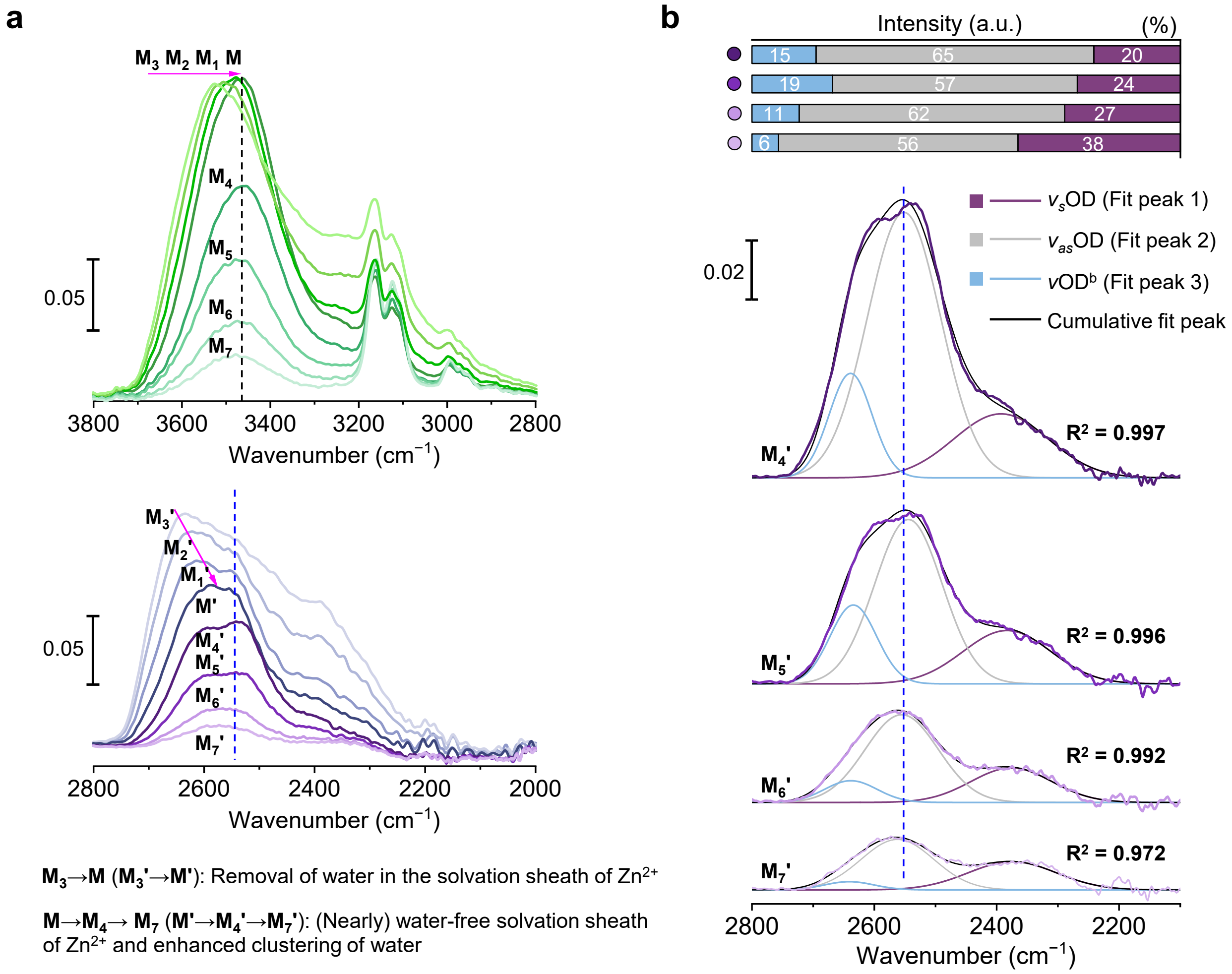


**Figure S5. Hydroxyl stretching band of electrolytes M→$M_7$ and M'→$M_7$'.** (a) Enlarged views of the O–H stretching band of electrolytes **M→$M_7$** and the O–D stretching band for electrolytes **M'→$M_7$'**. (b) Deconvolution of the O–D stretching band of various electrolytes **$M_4$'→$M_7$'** in the 2800–2100 $cm^{-1}$ range using Gaussian function, with the area percentages of three sub-bands inside the O–D stretching band presented in the bar chart. The spectra are offset for clarity. Vertical scale bars indicate the intensity of absorbance. The coefficient of determination ($R^2$) values are < 0.995 for electrolytes **$M_6$'** and **$M_7$'**, attributable to the elevated noise levels as peak intensity decreases.

**Note S7. Comparison of MD-simulated and FTIR-derived numbers of $H_2O$ in the primary solvation sheath of $Zn^{2+}$ as a function of electrolyte formulation.**

MD simulations on eight electrolytes **M**→**M**$_7$ were conducted to obtain their simulated liquid structures (Figure S6). The variation trend of water population in the primary solvation sheath of $Zn^{2+}$ (indicated by the CN of $Zn^{2+}$-O ($H_2O$) calculated from RDF results) in response to the change of compositional ratio exhibits strong consistency with the conclusions derived from the FTIR spectra (Figure S7, the FTIR-derived numbers of $H_2O$ in the primary solvation sheath of $Zn^{2+}$ are estimated by multiplying the combined fractional area of $\nu OH^{b}$ and $\nu OH^{f}$ bands by the average water-to-Zn salt ratio of the electrolyte): (1) as the formulation changes from **M**$_3$ to **M** ($n$ = 10 → 4), the CN of $Zn^{2+}$-O($H_2O$) decreases from 1.13 to 0.39, indicating the continuous removal of $H_2O$ in the $Zn^{2+}$ solvation sheath; (2) the solvation sheath of $Zn^{2+}$ is consistently dominated by $TFSI^{-}$ with the incremental addition of EmimTFSI ($x$ = 2 → 32 for electrolytes **M**→**M**$_4$→**M**$_7$), showing the CN of $Zn^{2+}$-O($H_2O$) of $\leq 0.21$; (3) clustering of $H_2O$ to yield nanoscale heterogeneity is thermdynamically favorable under conditions of low water concentrations for electrolytes **M**→**M**$_7$.

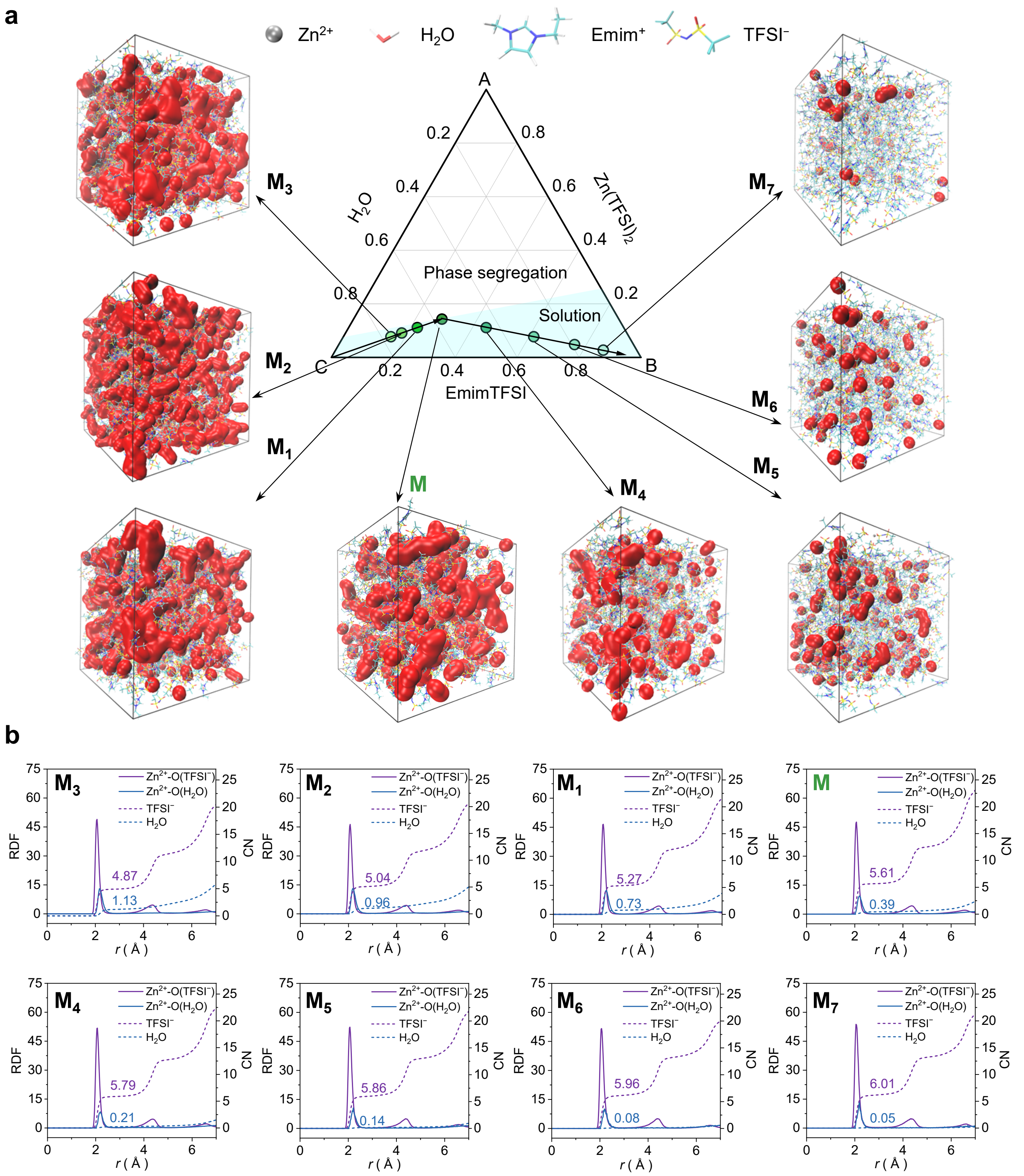


**Figure S6. Simulated liquid structures**. (a) Representative 3D snapshots and (b) corresponding RDFs of $Zn^{2+}$-O($TFSI^-$) and $Zn^{2+}$-O($H_2O$) as well as CNs of $TFSI^-$ and $H_2O$ obtained from MD simulations of electrolytes **M→M$_7$** at 303 K.

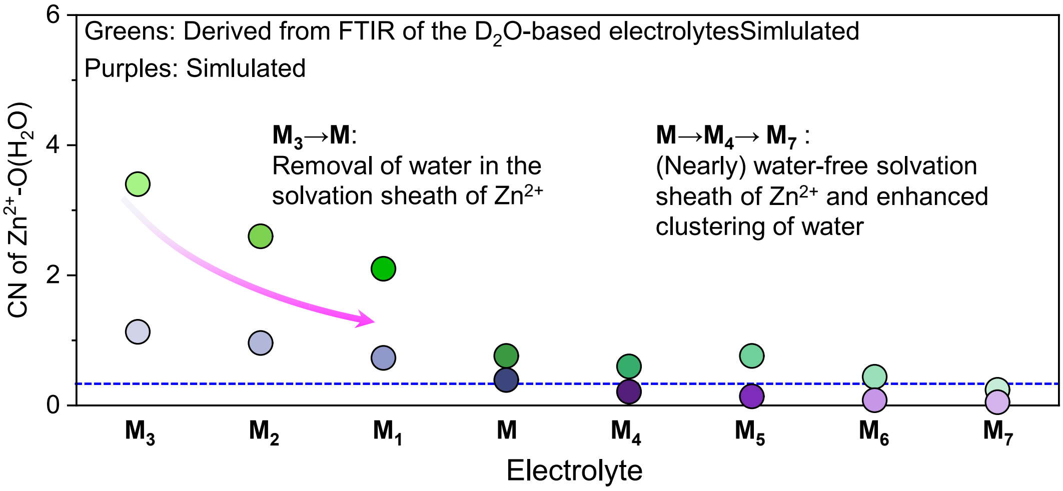


**Figure S7.** Comparison of MD-simulated and FTIR-derived numbers of $H_2O$ in the primary solvation sheath of $Zn^{2+}$ as a function of electrolyte formulation.

**Note S8. Correlating water $^1H$ chemical shift to Hofmeister effect in various Zn electrolytes.**

Zn salts with anions featuring high chaotropicity such as $TFSI^-$ and $ClO_4^-$ typically lead to the stronger shielding effect of hydrogen nuclei (large $\Delta\delta^1H$)[17, 18], while $Cl^-$ which has negligible Hofmeister effect (considered as a reference point in the Hofmeister series) marginally varies the electron density around the hydrogen nuclei ($\Delta\delta^1H = 0.08$ ppm for 1 m $ZnCl_2$)[19]; those kosmotropic anions like $SO_4^{2-}$ cause deshielding of the water hydrogen nuclei, thus a negative $\Delta\delta^1H$ value of −0.08 ppm is observed[20]; $\Delta\delta^1H$ of 4 m $Zn(TFSI)_2$ is 0.40 ppm, nearly 2.9 times that of 1 m $Zn(TFSI)_2$. Also using $Zn(TFSI)_2$ as the Zn salt, contrastingly, $^1H$ resonance of water molecules in $Zn(Emim)_2(TFSI)_4\cdot 4H_2O$ (electrolyte **M**) is detected with a quite negative $\Delta\delta^1H$ value of −0.10 ppm, indicating that the cation $Emim^+$, historically considered more likely to be a chaotrope in limited literature, here functions as a strong kosmotrope. Encouragingly, this abnormal mechanism can be once again rationalized by our assumption that, in electrolyte **M**, water molecules excluded from the primary solvation sheath of $Zn^{2+}$ are hydrogen-bonded to the $Emim^+$ and $TFSI^-$ outside with their hydrogen nucleus showing a lower electron density (deshielded).

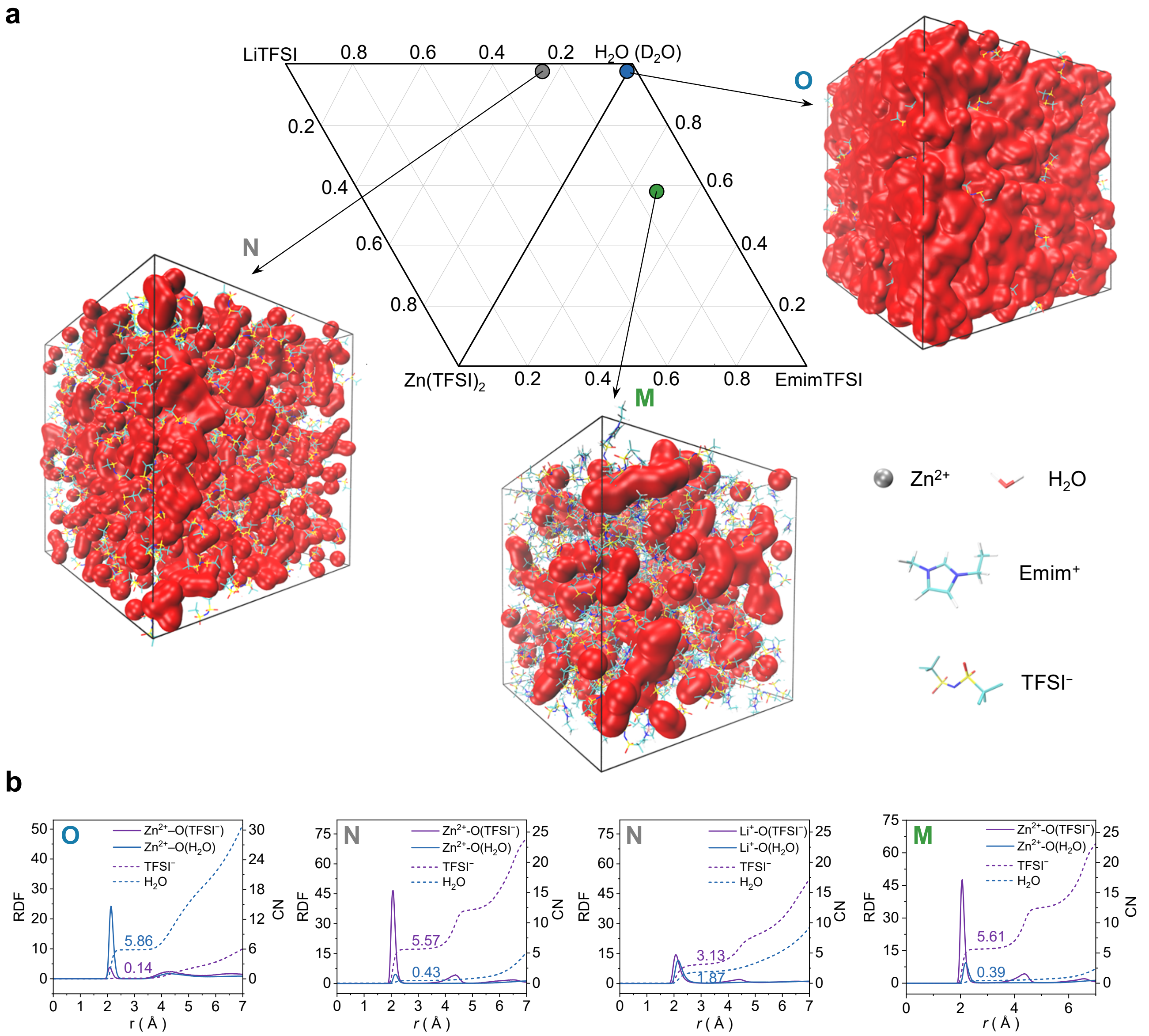


**Figure S8. Simulated liquid structures**. (a) Representative 3D snapshots and (b) corresponding RDFs of $Zn^{2+}$–O($TFSI^-$) and $Zn^{2+}$–O($H_2O$) as well as CNs of $TFSI^-$ and $H_2O$ obtained from MD simulations of electrolytes **O**, **N**, and **M** at 303 K.

**Note S9. Multispectroscopic analysis summary for elucidating the liquid structures.**

We introduce a workflow diagram (Figure S9) that synthesizes stepwise inferences from NMR, FTIR, Raman, ESI-MS, and SAXS data to the resolved liquid structures of the electrolytes under investigation. Specifically, (1) the prevalence of Emim–TFSI and Zn–$H_2O$ interactions was estimated by $^1$H NMR of C2–$\underline{H}$ and $\underline{H}_2O$; (2) to quantify the approximate number (prevalence) of water molecules in the solvation sheath of $Zn^{2+}$ ions in electrolyte **M**→**M$_7$**, subsequently, the hydroxyl stretching vibration band in the FTIR spectra was deconvoluted, with each of the four sub-bands distinctively attributed to a specific water state ($\nu_s$OH, $\nu_{as}$OH, $\nu$OH$^b$-$Zn^{2+}$, and $\nu$OH$^f$-$Zn^{2+}$) by including extensive reference samples and comprehensive literature-derived benchmarks; (3) by deconvoluting the S–N–S bending vibration in the Raman spectra into two modes respectively assigned to free and bonded TFSI, the Zn–TFSI binding affinity in different electrolytes was elucidated; (4) ESI-MS was applied to provide proxies for the relative strength of various intermolecular interactions. For example, the predominant $[(Emim)_2TFSI]^+$ species detected for electrolyte **M** implies favored clustering of $Emim^+$ with $TFSI^-$ over $Zn^{2+}$–$TFSI^-$ coordination; (5) synchrotron SAXS helps to determine the characteristic intermolecular distance of TFSI in varying local chemical environments and other $TFSI^-$-related microstructural characteristics (e.g., nanoscale heterogeneity). The nanostructures of electrolytes were eventually mapped by integrating all these clues.

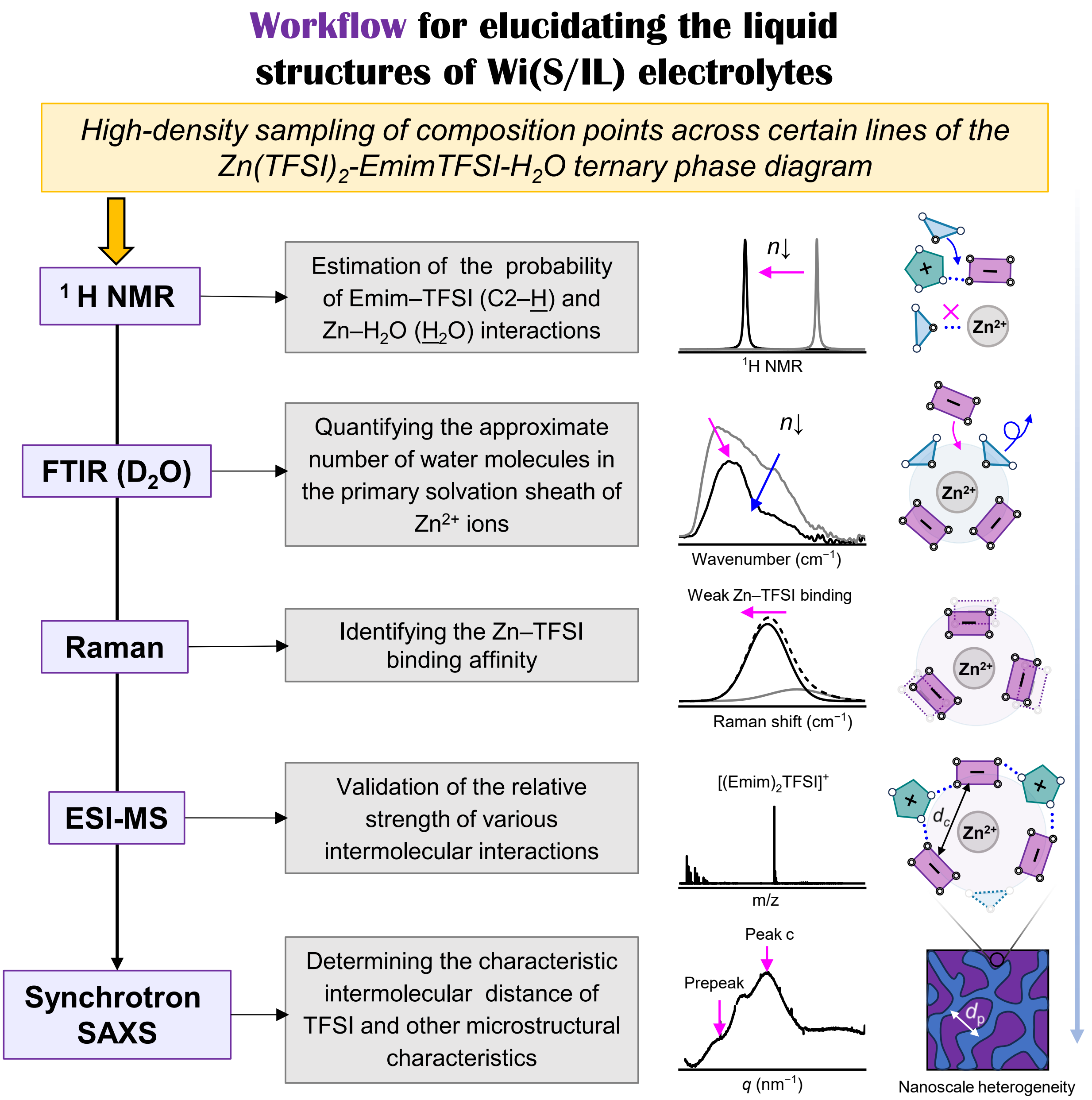


352

353 **Figure S9.** Schematic workflow for elucidating the liquid structures of the $Zn(TFSI)_2$-EmimTFSI-$H_2O$

354 ternary electrolytes.

355

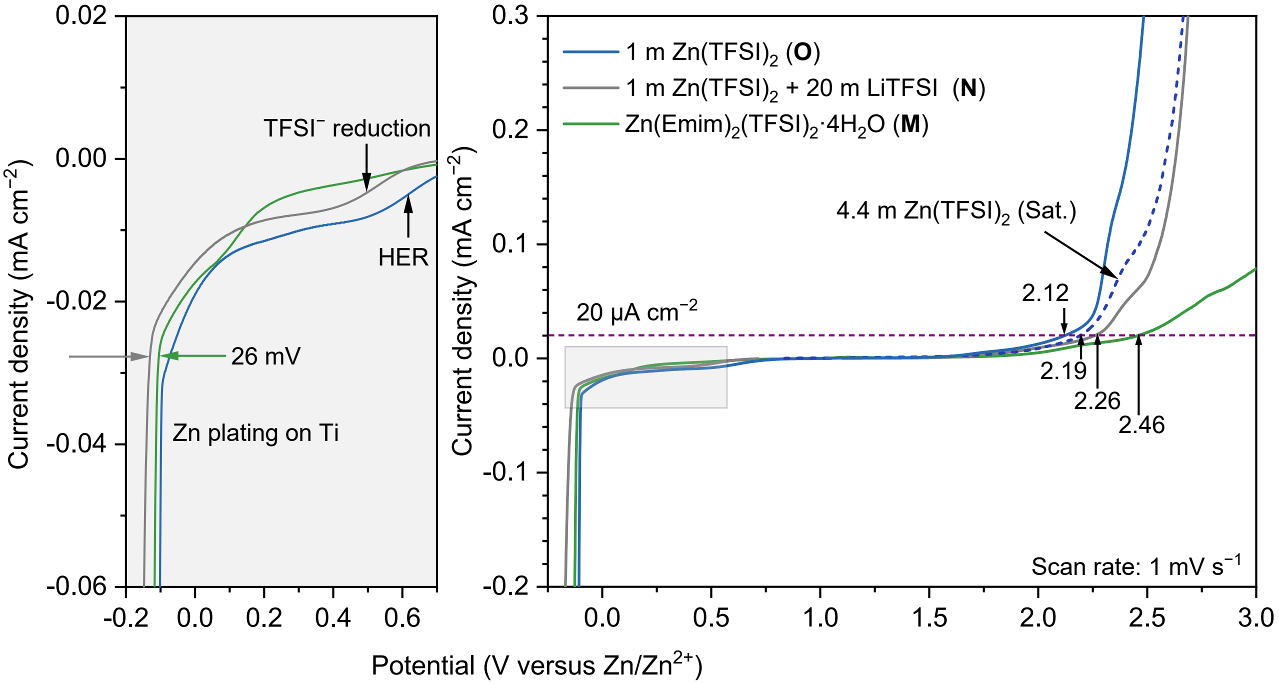


**Figure S10.** Linear sweep voltammetry (LSV) profiles of electrolytes measured in Zn||Ti two-electrode cells at a scan rate of 1 mV $s^{-1}$. The dashed horizontal line representing a cut-off current density of 20 μA $cm^{-2}$ indicates the anodic limit of electrolytes. The anodic LSV profile of saturated $Zn(TFSI)_2$ aqueous electrolyte is included to highlight the extended anodic limit of the WiS electrolyte and our proposed Wi(S/IL) electrolyte. Possible parasitic reactions before Zn plating on Ti are analyzed from the zoomed-in view of the cathodic scan.

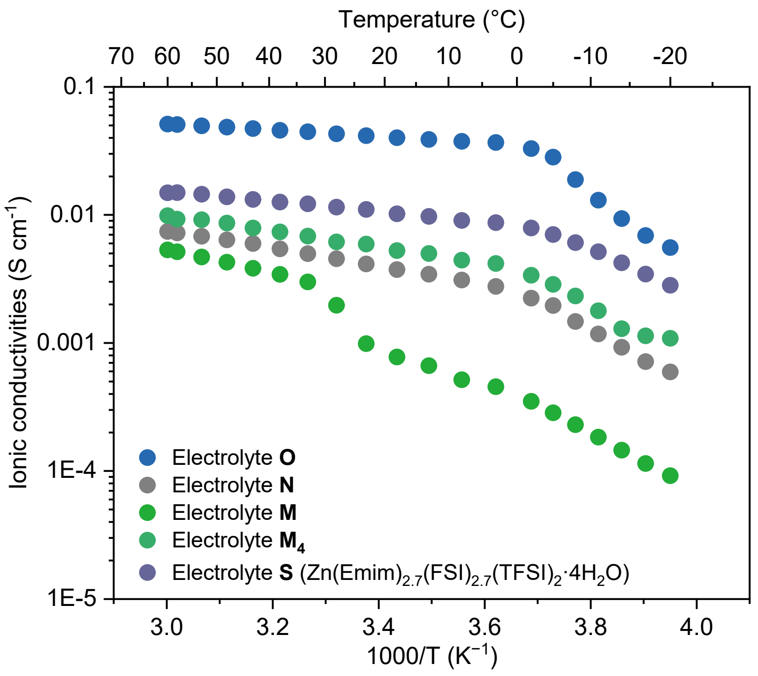


**Figure S11.** Arrhenius plots of ionic conductivity versus temperature for electrolytes **O**, **N**, **M**, **M$_4$**, and **S** within the temperature range of −20 to 60 °C. The ionic conductivity was measured using a conductivity meter.

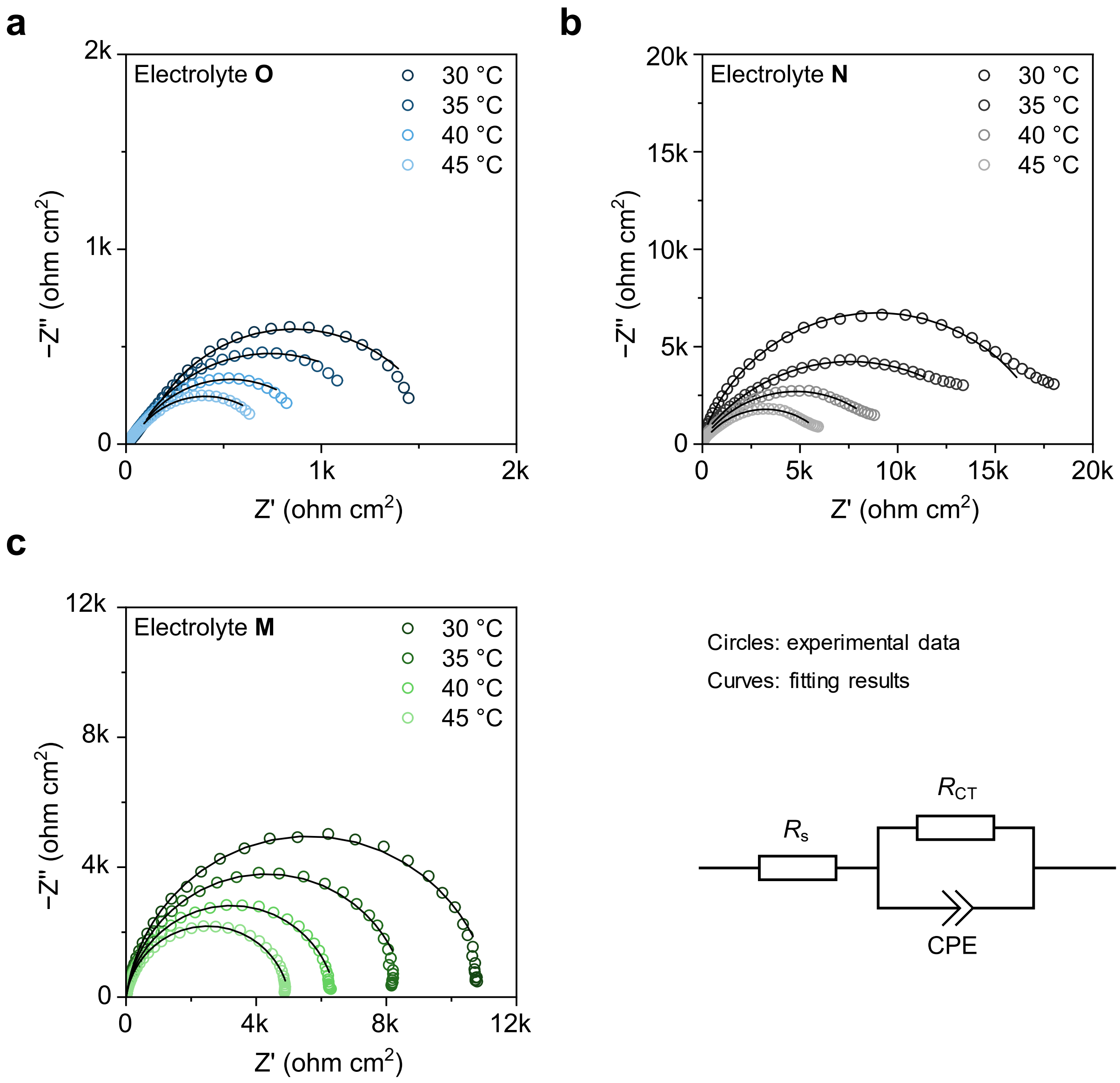


**Figure S12.** EIS fitting of charge transfer resistance in Zn||Zn symmetric cells in electrolytes **O** (a), **N** (b), and **M** (c) at temperatures from 30 to 45 °C with a 5 °C increment. The data were fitted (shown by the solid curves) using the Z-view program using the equivalent circuit proposed.

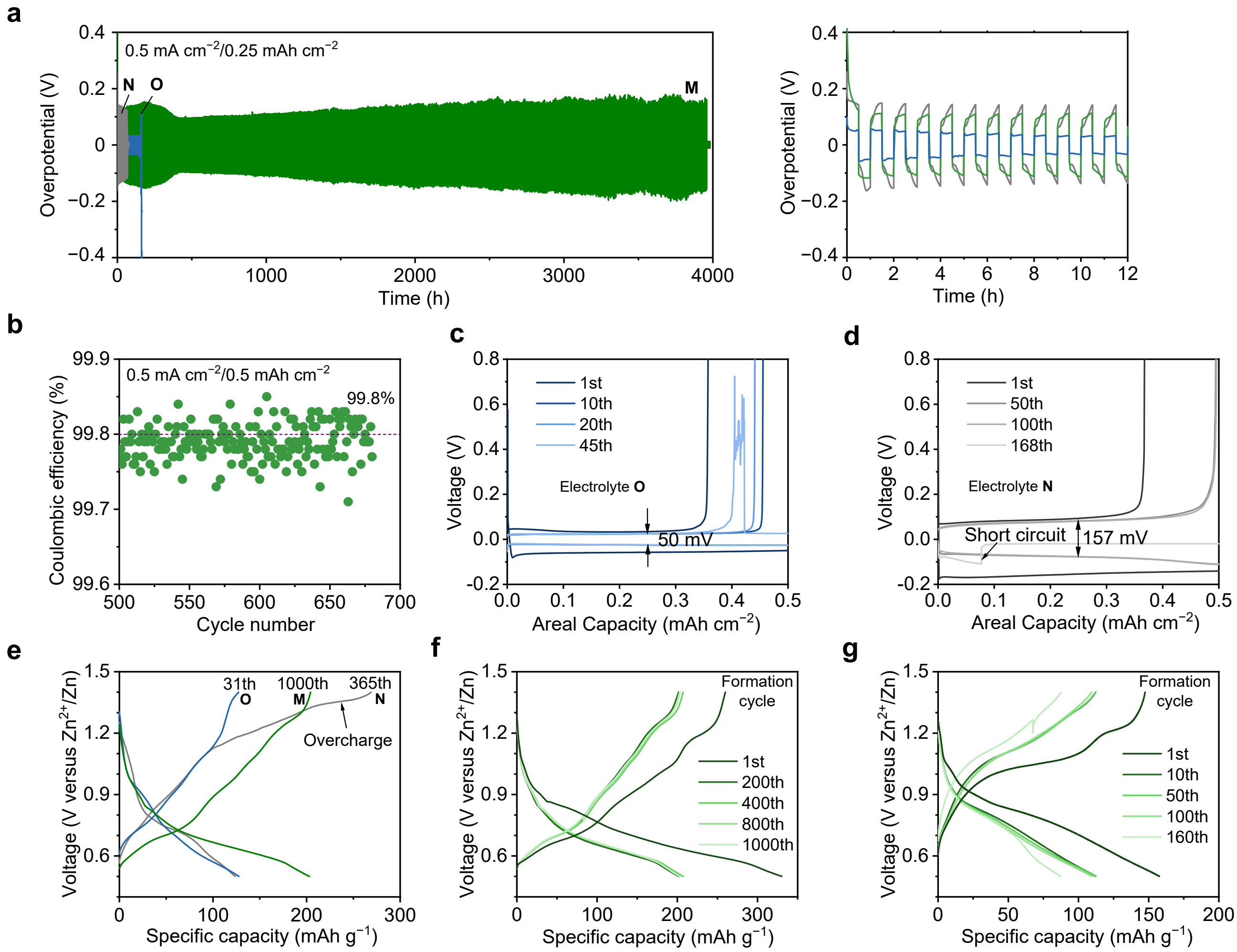


**Figure S13. Zn cycling performance of electrolytes in different cell configurations.** (a) Galvanostatic Zn plating/stripping in Zn||Zn symmetrical cells with electrolytes **O**, **N**, and **M** at 0.5 mA cm$^{-2}$ and a capacity of 0.25 mAh cm$^{-2}$. The right panel shows a zoomed-in view of the initial stage. (b) A zoomed-in view of CEs obtained in Zn||Cu asymmetrical cells with electrolyte **M**. Zn plating/stripping voltage profiles of the Zn||Cu asymmetrical cells cycled with electrolyte **O** (c) and **N** (d). All Zn||Cu cells were tested at 0.5 mA cm$^{-2}$/0.5 mAh cm$^{-2}$. The representative overpotential for these three electrolytes follows the order **O** < **M** < **N**. (e) Charge/discharge voltage profiles of a Zn||ZVO half cell at 31th, 365th, and 1000th cycle for O, N, and M, respectively. Overcharge due to "soft short" was observed for the cell with electrolyte **N**. (f) Charge/discharge voltage profiles (two formation cycles at 0.2 C, then 0.5 C) of a Zn||ZVO half cell with electrolyte **M** as a function of cycle number. (g) Charge/discharge voltage profiles (two formation cycles at 0.1 C, then 0.2 C) of a Zn (10 μm) ||ZVO (20 mg cm$^{-2}$) full cell with electrolyte **M** as a function of cycle number (1 C = 6 mA cm$^{-2}$).

**Note S10. Studies on long-term cyclability, rate capability, storage performance, and operability at extreme temperature conditions.**

The storage performance of electrolyte **M** evaluated by CE is overwhelmingly better than that of electrolytes **O** and **N**, additionally, the typical average discharge voltage is only slightly lower than that achieved without resting (0.656 versus 0.676 V) despite the occurrence of faster voltage decay (Figure S14e), which indicates a limited energy loss after the long-term storage (evaluating the self-discharge rate based on the decay rate of cell's open circuit potential may provide a misleading result here). Very few works have reported on durable aqueous Zn battery chemistries at temperatures higher than 70 °C.[21-25] Herein, a cycling life of ~200 cycles (80% retention) and a cyclability of 720 cycles at temperatures beyond 70 °C are, to our best knowledge, never reported before (Figure S14f,g). The temperature-dependent cycling stability test results show electrolyte M can extend the upper limit of operation temperature to 100 °C; concurrently, at the low temperature end of −20 °C, an acceptable specific capacity of ca. 50 mAh $g^{-1}$ at 0.2 C rate is achieved (Figure S14h,i). Such an impressively wide operation temperature range of −20–100 °C of Zn storage chemistry afforded by electrolyte **M** is definitely among the best-performing aqueous electrolytes.[26] Additionally, with Electrolyte M, a Zn||ZVO full cell featuring a limited Zn source (10 μm) and a high-mass-loading ZVO cathode (20 mg $cm^{-2}$) achieved stable cycling for over 250 cycles at 0.5 C (3 mA $cm^{-2}$), maintaining a discharge capacity of ~60 mAh $g^{-1}$. GITT analysis reveals that the capacity limitation shifts from electrolyte ion transport at low rates to cathode solid-state diffusion at high rates.

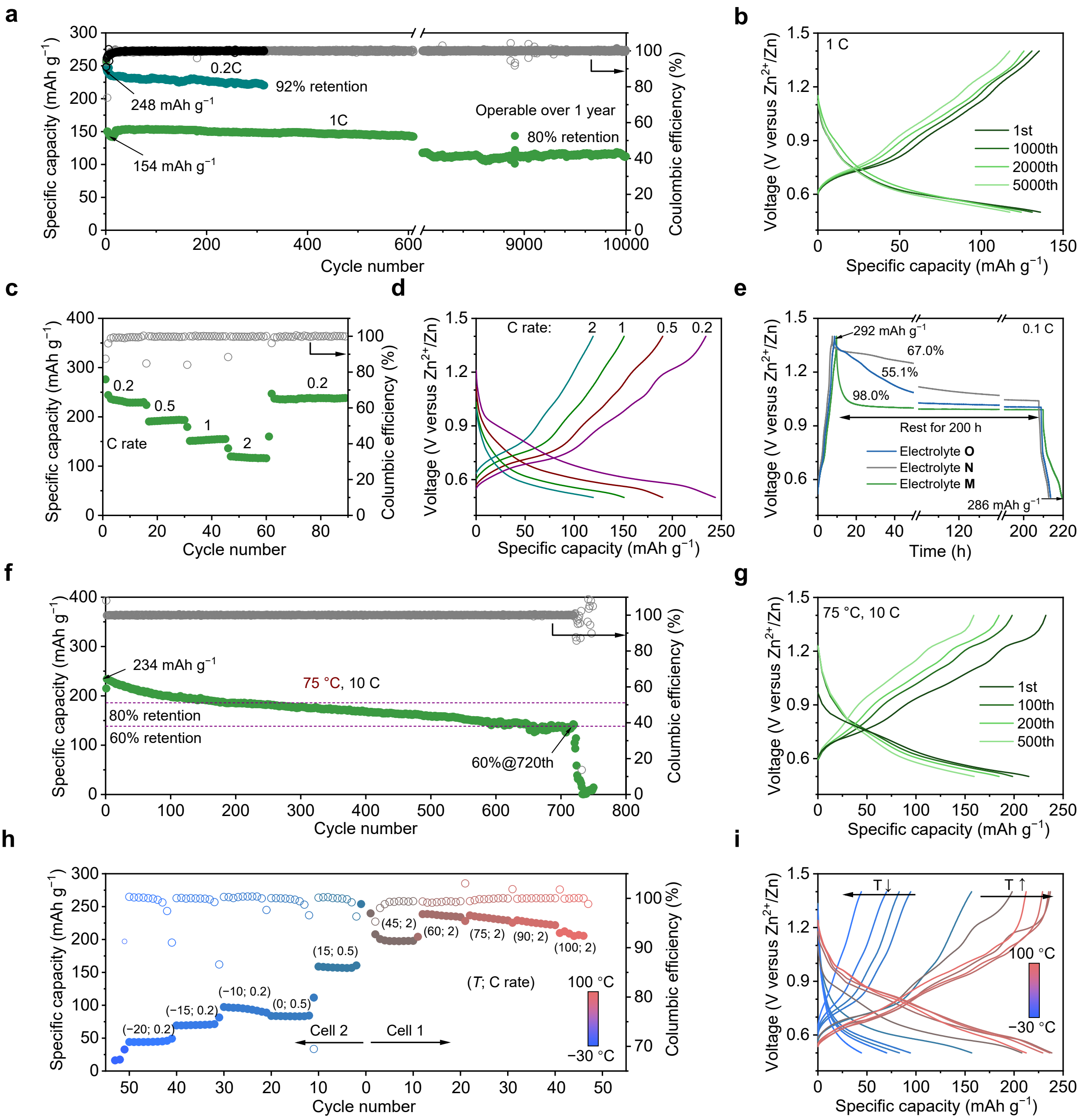


**Figure S14. Zn||ZVO cycling performance of electrolyte M under different test conditions.** Capacity retention at 0.2 C and 1 C rates (a) and corresponding 1 C rate charge/discharge voltage profiles (b) as a function of cycle number. Rate capability (c) and charge/discharge voltage profiles at different C rates (d). (e) Storage performance comparison evaluated by resting cells with different electrolytes at 100% state of charge (SOC) for 200 h followed by full discharge. Capacity retention at 75 °C and 10 C rate (f) and corresponding charge/discharge voltage profiles (g) as a function of cycle number. Variable-temperature cycling performance (h) and charge/discharge voltage profiles at each temperature (i). Two fresh cells were respectively subjected to the heating up procedure from 45 to 100 °C (Cell 1) and the cooling down procedure from 15 to −30 °C (Cell 2).

**Note S11. General applicability of the Wi(S/IL) strategy.**

Following the Wi(S/IL) design principle, we randomly formulated four additional electrolytes (making a total of five, including the previously studied electrolyte $\mathbf{M_4}$) and characterized their fundamental electrochemical properties. The results show that, except for $Zn(Emim)_{1.0}(OAc)_{2.0}(DCA)_{1.0}\cdot 4H_2O$, the other electrolytes combine relatively high ionic conductivity with excellent electrochemical stability. Zn plating/stripping tests revealed that all four new electrolytes enable CEs exceeding 99.5%. The cell employing $ZnCl_2$-based Wi(S/IL) electrolyte failed after a limited number of cycles, which is likely attributable to corrosion of coin-cell components by $Cl^-$ rather than an inherent flaw in the electrolyte design. The cell with $Zn(Emim)_{4.0}(OTf)_{6.0}\cdot 8H_2O$ sustained stable cycling for over 250 cycles with an average CE of ~99.4%. Notably, electrolyte $\mathbf{M_4}$ demonstrated performance comparable to that of Electrolyte **M**. More impressively, Electrolyte S—obtained by replacing EmimTFSI in Electrolyte M with an equivalent amount of EmimFSI—further boosted the stable cycling Coulombic efficiency to 99.9%, with an average exceeding 99.85%. To our knowledge, this places it among the best-performing electrolytes for aqueous Zn batteries. The rate capability of electrolyte **S** was further evaluated at different current densities from 0.2 to 50 C. At C rates below 10 C, this electrolyte afforded stable specific capacities of > 160 mAh $g^{-1}$ and within this rate range the capacity decreased marginally with C rate. Remarkably, even at very challenging current densities of up to 50 C (corresponding to a current density of 30 mA $cm^{-2}$), reasonable specific capacities of > 60 mAh $g^{-1}$ along with typical battery-type charge/discharge voltage profiles were observed (Figure S15a, b). Such high-rate performances even outperform that of the $V_2O_5$@PEDOT cathode operated with a conventional aqueous electrolyte of 2 M $Zn(OTf)_2$[27], demonstrating that the Wi(S/IL) electrolytes do not necessarily lead to lower specific capacity. The long-term cycling test was performed at 5 C after 5

formation cycles, as seen, after the initial stage where the capacity experiences a gradual increase process to reach the plateau value of 185 mAh $g^{-1}$, no obvious capacity decay is observed before a soft short circuit at $1815^{th}$ cycle (Figure S15c). The high reversibility of electrode reaction and interfacial stability afforded by the investigated electrolyte were further indicated by the nearly overlapped charge-discharge voltage profiles (Figure S15d).

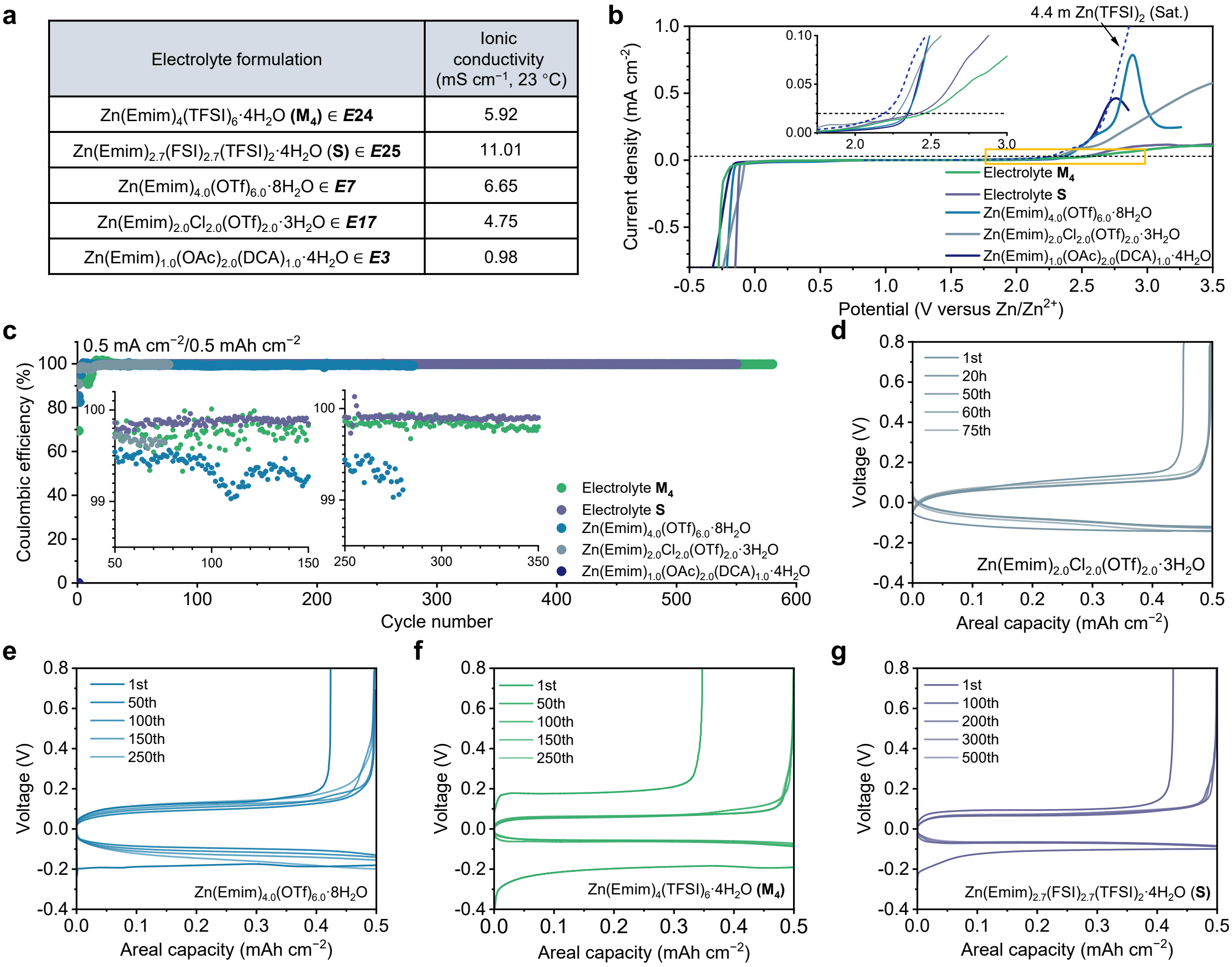


| Electrolyte formulation | Ionic conductivity (mS cm$^{-1}$, 23 °C) |
|---|---|
| $Zn(Emim)_4(TFSI)_6\cdot 4H_2O$ (**$M_4$**) ∈ ***E24*** | 5.92 |
| $Zn(Emim)_{2.7}(FSI)_{2.7}(TFSI)_2\cdot 4H_2O$ (**S**) ∈ ***E25*** | 11.01 |
| $Zn(Emim)_{4.0}(OTf)_{6.0}\cdot 8H_2O$ ∈ ***E7*** | 6.65 |
| $Zn(Emim)_{2.0}Cl_{2.0}(OTf)_{2.0}\cdot 3H_2O$ ∈ ***E17*** | 4.75 |
| $Zn(Emim)_{1.0}(OAc)_{2.0}(DCA)_{1.0}\cdot 4H_2O$ ∈ ***E3*** | 0.98 |

**Figure S15. A collection of the basic electrochemical properties and Zn deposition/dissolution performance of five electrolytes belonging to different Wi(S/IL) electrolyte systems.** (a) Ionic conductivity at 23 °C. (b) LSV profiles measured in Zn||Ti two-electrode cells at a scan rate of 1 mV s$^{-1}$. (c) Zn plating/stripping CE in Zn||Cu cells at 0.5 mA cm$^{-2}$/0.5 mAh cm$^{-2}$ and (d–g) the corresponding charge/discharge voltage profiles as a function of cycle number.

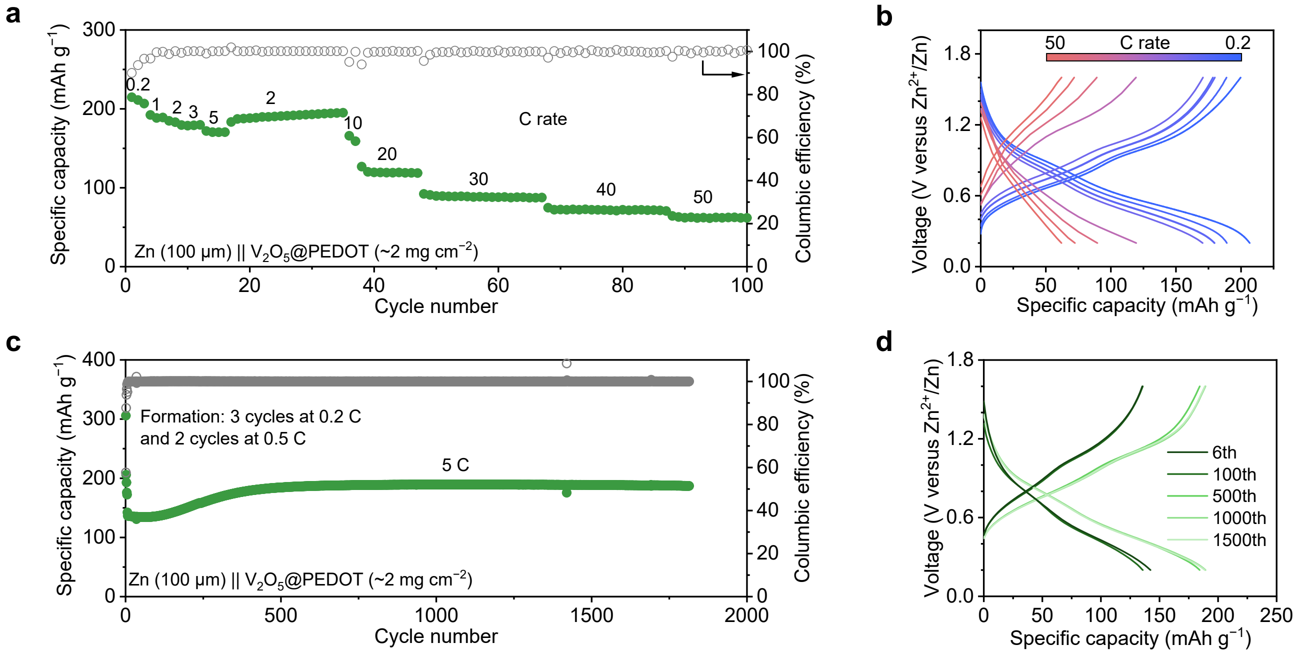


**Figure S16. Cell performance of Zn||$V_2O_5$@PEDOT with Zn(Emim)$_{2.7}$(FSI)$_{2.7}$(TFSI)$_2$·4$H_2O$.** Rate capability (a) and representative charge/discharge voltage profiles at different current densities up to 50 C rate (based on $V_2O_5$@PEDOT, 1 C = 300 mA $g^{-1}$ ≈ 0.6 mA $cm^{-2}$) (b). Capacity retention at 5 C rate (c) and corresponding charge/discharge voltage profiles (d) as a function of cycle number.

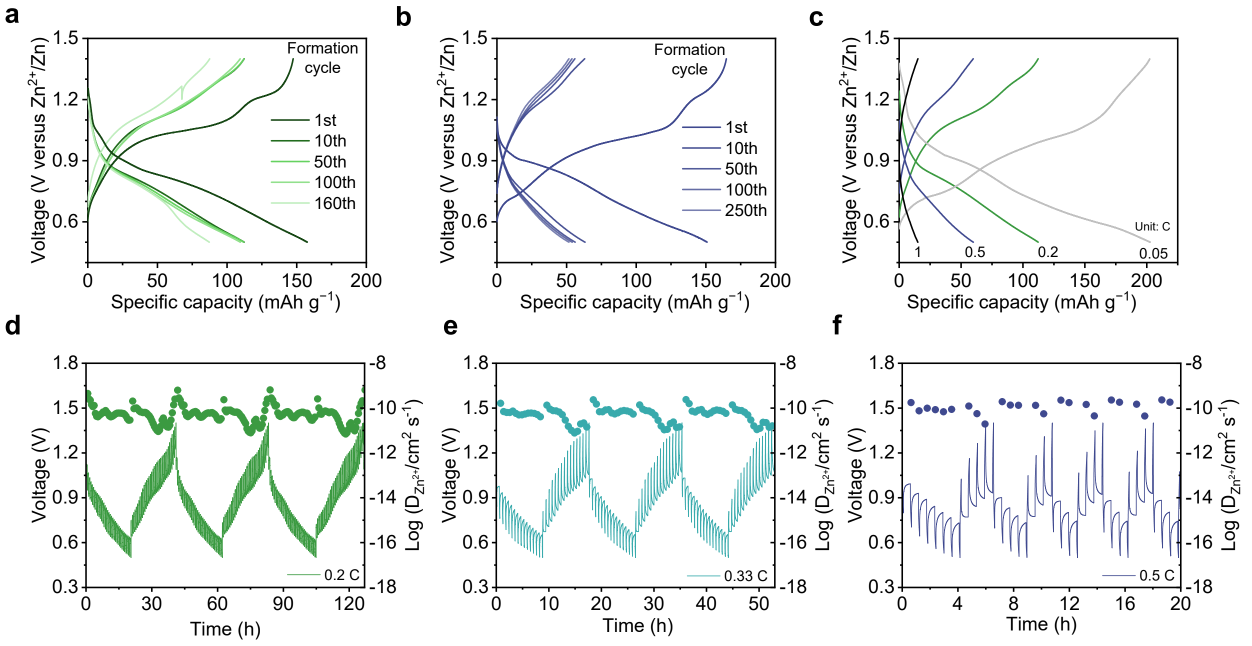


**Figure S17. Charge/discharge voltage profiles of Zn||ZVO full cells with electrolyte M.** Voltage profiles as a function of cycle number at 0.2 C (a) and 0.5 C (b). C rate-dependent voltage profiles. (d–f) Voltage profiles obtained via galvanostatic intermittent titration technique (GITT) measurements at different C rates. GITT analysis reveals that the capacity limitation shifts from ion transport in the electrolyte at low rates to cathode solid-state diffusion at higher rates.

**Note S12. Additional discussions on Zn cycling interphasial chemistry.**

The back scattered electron (BSE) image and wavelength dispersive spectroscopy (WDS) elemental maps of the cycled Zn reveal the absence of either dendritic Zn or Zn texturing behavior, instead, the Zn reaction layer is seemingly pulverized and mainly covered with polymeric species abundant in C, S, and F elements (**Fig. 5a**). From the XRD pattern of cycled Zn (Figure S16d), the intensity ratio of Zn(002) to Zn(100) is calculated to be 1.6:1, close to that of pristine Zn, showing no texturing behavior. New peaks at 31.80°, 34.43°, 47.47°, 63.98°, and 67.96° can be indexed to (100), (002), (102), (103), and (112) diffraction planes of ZnO (PDF#01-1136), and the remaining new peak at 56.59° is assigned to (311) plane of ZnS (PDF#05-0566). This result agrees well with the TEM results that ZnO is the dominant inorganic species in both the outer polymeric layer and the surface of the Zn substrate. ZnS shows the second-highest abundance, but it only exists in the polymeric layer. $Zn(OH)_2$ and other possible reduction products, such as $ZnCO_3$ and $ZnSO_3$, are very limited. Depth-profiling XPS results show that the polymeric species, indicated by peaks attributed to $*CF_3$/$*SO_2CF_3$ in spectra of C1 s, O 1s, S 2p, and F 1s, distribute uniformly along the depth direction, further evidencing that the micro-sized Zn deposits are separately covered by the polymeric species to form the unique deposition layer as the schematic shows.

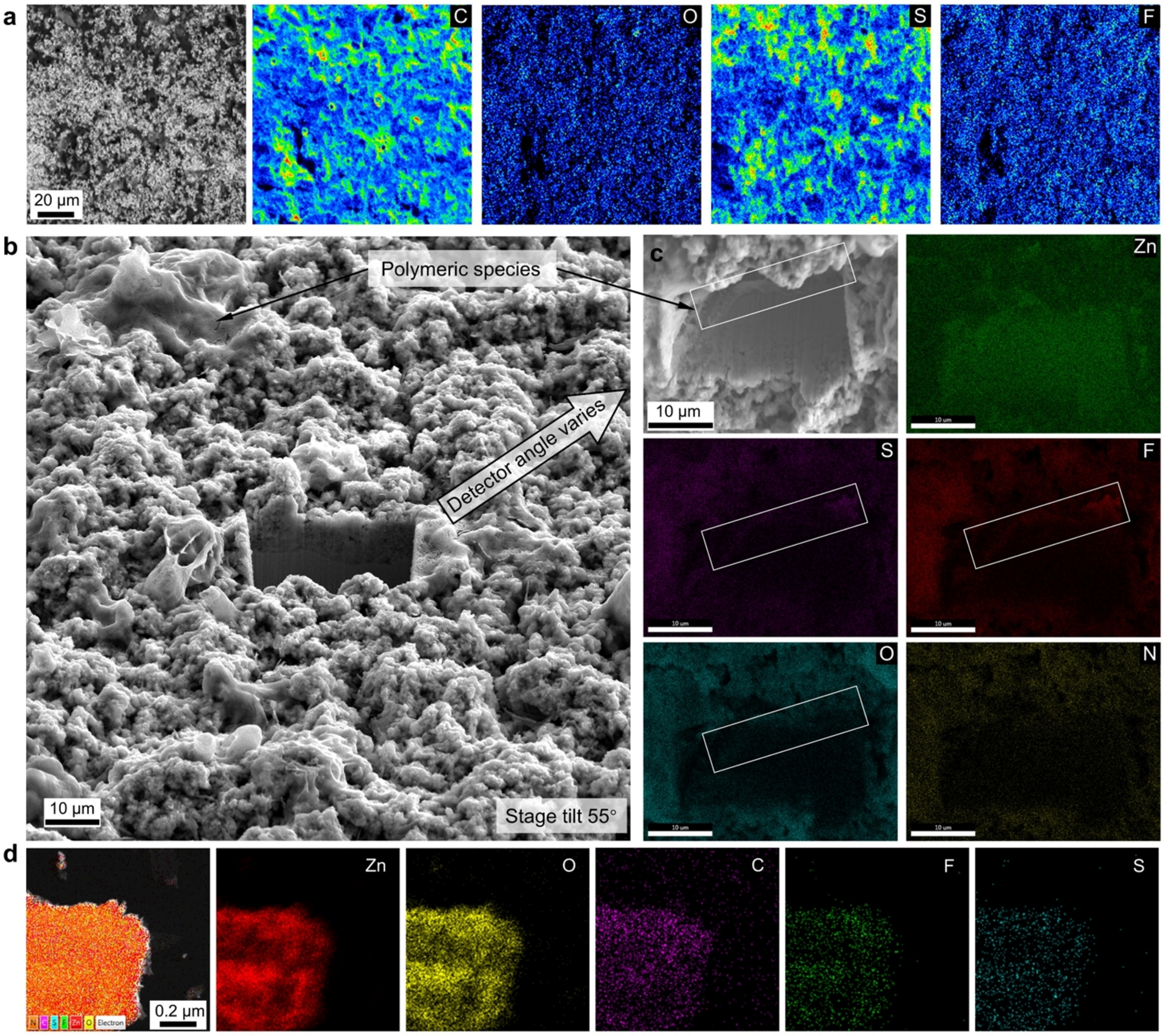


**Figure S18. Morphological and compositional analyses on the deposition layer of Zn cycled with electrolyte M.** (a) BSE image and WDS elemental maps of C, O, S, and F. (b) Low-magnification SEM image indicating the prevailing presence of polymeric species on the surface. No obvious protuberance is observed. (c) Cross-sectional SEM image and corresponding EDS elemental maps of Zn, S, F, O, and N obtained from a different angle. The white boxes highlight that the polymeric species are abundant in S, F, and O elements. (d) TEM-EDS elemental maps of a Zn deposit, Zn, O, C, F, S, and all elements overlaid in one image.

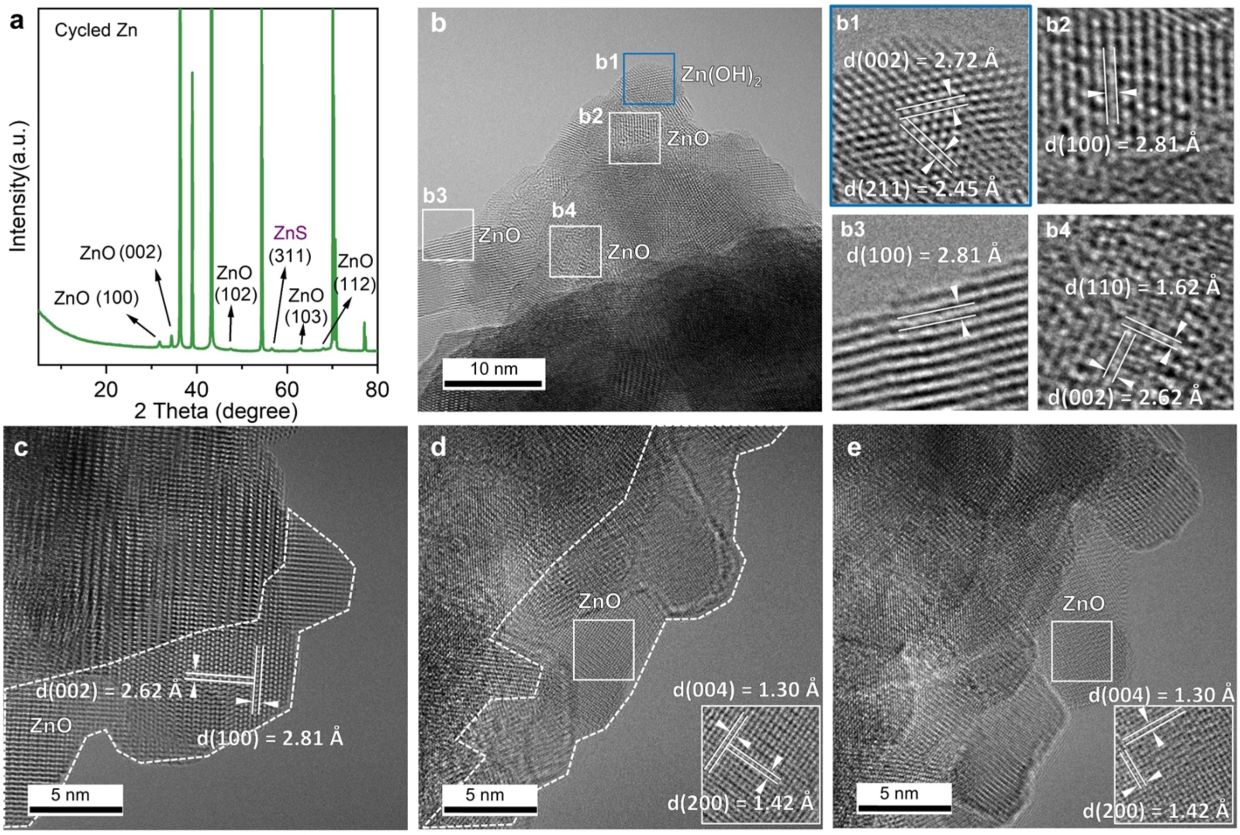


**Figure S19. Crystallographic and morphological analysis of zinc deposits cycled with electrolyte M.** (d) XRD pattern. (b–e) Atomic-resolution TEM images acquired from different regions of the bare Zn deposits.

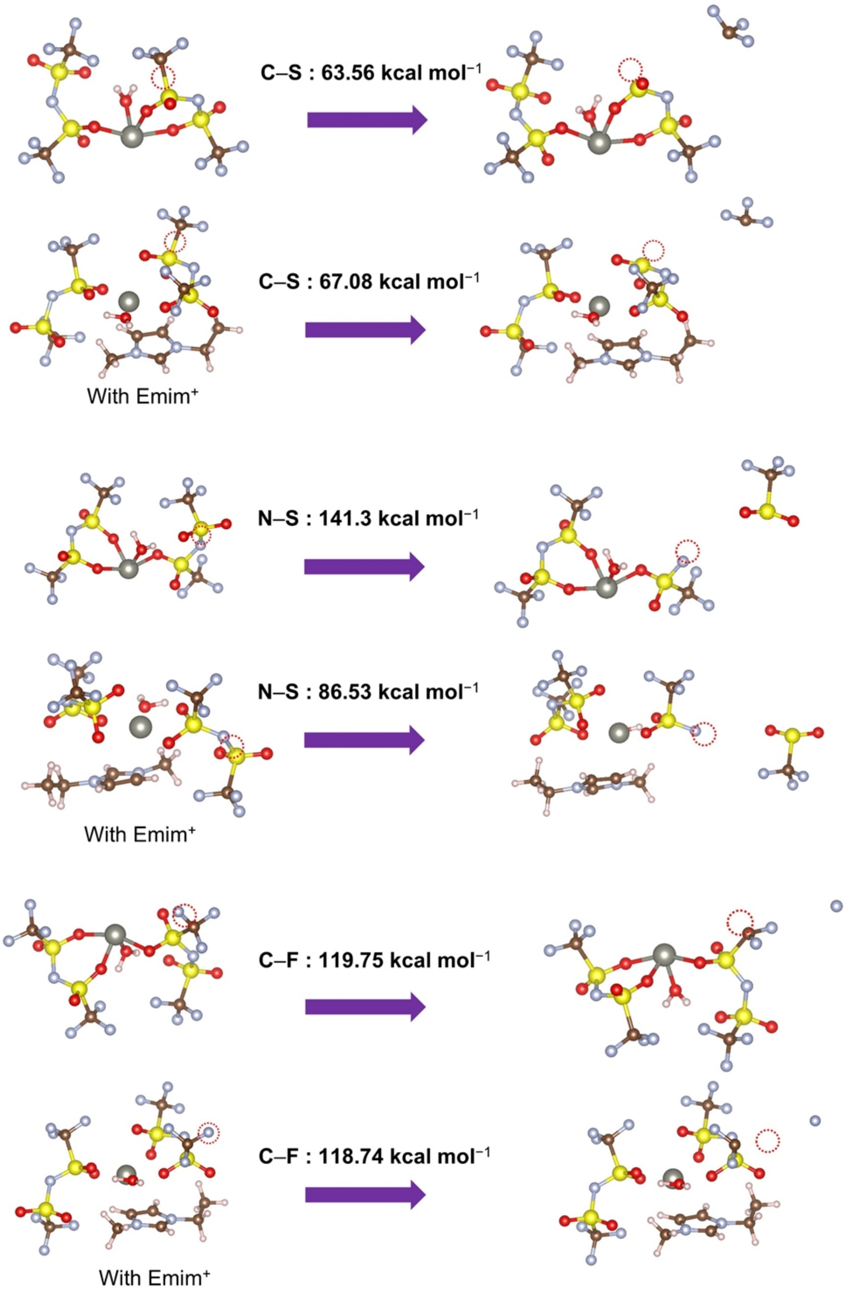


**Figure S20.** Calculated thermal BDEs for bond cleavage in TFSI$^-$ solvated to $Zn^{2+}$, without and with Emim$^+$.

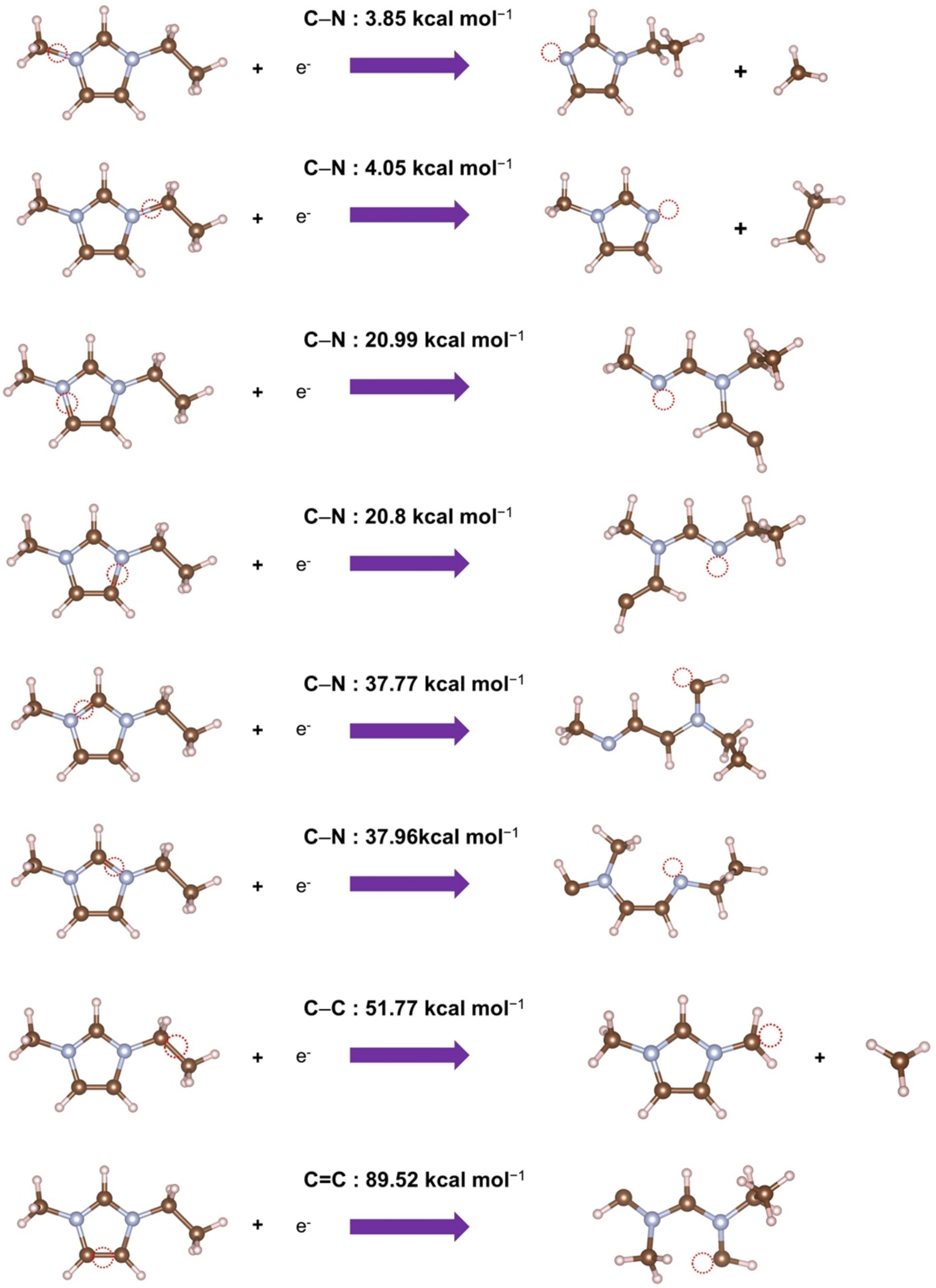


**Figure S21.** Calculation results of the BDEs for bond cleavage in $Emim^{+}$ upon cathodic reduction.

**Table S1.** Summary of electrolyte compositions investigated in this work.

| General formula | Composition / Molality, m | General formula | Composition / Molality, m |
|---|---|---|---|
| $Zn(Emim)_2(TFSI)_4{\cdot}4H_2O$ (**M**) | 13.9 m $Zn(TFSI)_2$ + 27.8 m EmimTFSI | $Zn(Emim)_{16}(TFSI)_{18}{\cdot}4H_2O$ (**$M_6$**) | 13.9 m $Zn(TFSI)_2$ + 222.2 m EmimTFSI |
| $Zn(Emim)_2(TFSI)_4{\cdot}6H_2O$ (**$M_1$**) | 9.3 m $Zn(TFSI)_2$ + 18.5 m EmimTFSI | $Zn(Emim)_{32}(TFSI)_{34}{\cdot}4H_2O$ (**$M_7$**) | 13.9 m $Zn(TFSI)_2$ + 444.4 m EmimTFSI |
| $Zn(Emim)_2(TFSI)_4{\cdot}8H_2O$ (**$M_2$**) | 6.9 m $Zn(TFSI)_2$ + 13.9 m EmimTFSI | $Zn(Emim)_{2.7}(FSI)_{2.7}(TFSI)_2{\cdot}4H_2O$ (**S**) | 13.9 m $Zn(TFSI)_2$ + 37.2 m EmimFSI |
| $Zn(Emim)_2(TFSI)_4{\cdot}10H_2O$ (**$M_3$**) | 5.6 m $Zn(TFSI)_2$ + 11.1 m EmimTFSI | $Zn(Emim)_{1.0}(OAc)_{2.0}(DCA)_{1.0}{\cdot}4H_2O$ | 13.9 m $Zn(OAc)_2$ + 13.9 m EmimDCA |
| $Zn(Emim)_4(TFSI)_6{\cdot}4H_2O$ (**$M_4$**) | 13.9 m $Zn(TFSI)_2$ + 55.6 m EmimTFSI | $Zn(Emim)_{4.0}(OTf)_{6.0}{\cdot}8H_2O$ | 6.9 m $Zn(OTf)_2$ + 27.8 m EmimOTf |
| $Zn(Emim)_8(TFSI)_{10}{\cdot}4H_2O$ (**$M_5$**) | 13.9 m $Zn(TFSI)_2$ + 111.1 m EmimTFSI | $Zn(Emim)_{2.0}Cl_{2.0}(OTf)_{2.0}{\cdot}3H_2O$ | 18.3 m $ZnCl_2$ + 36.7 m EmimOTf |

**Table S2**. Peak positions obtained with Gaussian fitting.

| Sample | Peak position ($cm^{-1}$) | | | |
|---|---|---|---|---|
| | $\nu_s$OD (Peak 1) | $\nu_{as}$OD (Peak 2) | $\nu OD^b$ (Peak 3) | $\nu OD^f$ (Peak 4) |
| Pure $D_2O$ | 2381 | 2516 | 2623 | 2684 |
| Electrolyte $\mathbf{M_3}$' | 2415 | 2542 | 2623 | 2678 |
| Electrolyte $\mathbf{M_2}$' | 2411 | 2550 | 2629 | 2681 |
| Electrolyte $\mathbf{M_1}$' | 2416 | 2550 | 2630 | 2684 |
| Electrolyte **M**' | 2388 | 2563 | 2643 | 2679 |
| Electrolyte $\mathbf{M_4}$' | 2393 | 2551 | 2639 | — |
| Electrolyte $\mathbf{M_5}$' | 2383 | 2544 | 2634 | — |
| Electrolyte $\mathbf{M_6}$' | 2376 | 2555 | 2638 | — |
| Electrolyte $\mathbf{M_7}$' | 2375 | 2562 | 2641 | — |
| Electrolyte **O**' | 2400 | 2517 | 2617 | 2669 |
| Electrolyte **N**' | 2433 | 2548 | 2607 | 2687 |

**Table S3**. Comparison of Zn cycling reversibility reported in this work and the representative achievements reported in recent years (≥ 2022)[28–46].

| Electrolyte formulation | Reference | Averaged CE[a] | Stabilized CE[b] | Number of cycles | Testing protocol and cell configuration |
|---|---|---|---|---|---|
| $Zn(TFSI)_2$/EmimTFSI/$H_2O$ = 1/2/4 (mol/mol/mol) | **This work** | **99.7%** | **99.75%** | **680** | 0.5 mA $cm^{-2}$/0.5 mAh $cm^{-2}$, Zn\|\|Cu |
| $Zn(TFSI)_2$/EmimTFSI/$H_2O$ = 1/4/4 (mol/mol/mol) | | **99.7%** | **99.80%** | **> 580** | 0.5 mA $cm^{-2}$/0.5 mAh $cm^{-2}$, Zn\|\|Cu |
| $Zn(TFSI)_2$/EmimFSI/$H_2O$ = 1/2.7/4 (mol/mol/mol) | | **99.85%** | **99.90%** | **> 550** | 0.5 mA $cm^{-2}$/0.5 mAh $cm^{-2}$, Zn\|\|Cu |
| 2 M $ZnSO_4$ + 0.2 M EmimOAc | *Angew. Chem. Int. Ed.* ***2024****, 63, e202318470.* | 99.7% | N.A. | 1000 | 1.0 mA $cm^{-2}$/0.5 mAh $cm^{-2}$, Zn\|\|Cu |
| 2 M $ZnSO_4$ + 0.05 M BmimBr | *J. Am. Chem. Soc.* ***2025****, 147, 8523.* | 99.42% | N.A. | 2100 | 1.0 mA $cm^{-2}$/1.0 mAh $cm^{-2}$, Zn\|\|Cu |
| 2 M $Zn(OTf)_2$ + 0.5 M EmimOTf | *PNAS,* ***2024****, 121, e2317796121.* | 99.7% | N.A. | 2000 | 5.0 mA $cm^{-2}$/1.0 mAh $cm^{-2}$, Zn\|\|Cu |
| 2 m $Zn(OTf)_2$ in $H_2O$/EmimTFA (v/v =8:2) | *Nat. Commun.* ***2026****, 17, 736.* | 99.3 | N.A. | 1000 | 5.0 mA $cm^{-2}$/1.0 mAh $cm^{-2}$, Zn\|\|Cu |
| $Zn(TFSI)_2$ in FDMA/$H_2O$ | *J. Am. Chem. Soc.* ***2025****, 147, 2714.* | 99.4% | N.A. | 950 | 0.5 mA $cm^{-2}$/0.5 mAh $cm^{-2}$, Zn\|\|Cu |
| 2 M $Zn(OTf)_2$ in $H_2O$/EC (v/v =1:1) | *J. Am. Chem. Soc.* ***2025****, 147, 8607.* | 99.7% | N.A. | 2000 | 5.0 mA $cm^{-2}$/1.0 mAh $cm^{-2}$, Zn\|\|Cu |
| $Zn(OTf)_2$ in $H_2O$/EGME | *J. Am. Chem. Soc.* ***2024****, 146, 20508.* | N.A. | 99.9% | 500 | 1.0 mA $cm^{-2}$/1.0 mAh $cm^{-2}$, Zn\|\|Cu |
| 2 m $ZnSO_4$ + 0.1 m ASA | *Chem. Sci.* ***2024****, 15, 230.* | N.A. | 99.69% | 700 | 1.0 mA $cm^{-2}$/1.0 mAh $cm^{-2}$, Zn\|\|Ti |
| 1 M $Zn(OTf)_2$ in $H_2O$/methanol (mol/mol = 11:14) | *J. Am. Chem. Soc.* ***2023****, 145, 22456.* | 99.5% | N.A. | 300 | 1.0 mA $cm^{-2}$/1.0 mAh $cm^{-2}$, Zn\|\|Cu |

| | | | | | |
|---|---|---|---|---|---|
| 1 M $Zn(OTf)_2$ in $H_2O$/DMAC/TMP (v/v/v =5:2:3) | *Nat. Commun.* ***2023**, 14, 2720.* | 99.5% | N.A. | 2000 | 1.0 mA $cm^{-2}$/1.0 mAh $cm^{-2}$, Zn\|\|Cu |
| 2 m $Zn(OTf)_2$ in $H_2O$/SL (w/w = 3:7) | *Nat. Commun.* ***2023**, 14, 3067.* | 99.8% | 99.9 | 200 | 1.0 mA $cm^{-2}$/1.0 mAh $cm^{-2}$, Zn\|\|Cu |
| 30 m $ZnCl_2$+10 m TMACl+5 m LiCl in $H_2O$/DMC (mol/mol = 5:1) | *Nat. Sustain.* ***2023**, 6, 806.* | 99.95% | N.A. | 50 | 0.2 mA $cm^{-2}$, a modified galvanostatic technique |
| 10 m $Zn(Ac)_2$ + 15 m KAc | *Nat. Sustain.* ***2023**, 6, 1474.* | 99.4% | 99.6% | 300 | 0.25 mA $cm^{-2}$/0.25 mAh $cm^{-2}$, Zn\|\|Cu |
| 3 M $ZnSO_4$ + 5 vol% EGME | *Nano Lett.* ***2023**, 23, 541.* | N.A. | 99.5% | 600 | 2.0 mA $cm^{-2}$/1.0 mAh $cm^{-2}$, Zn\|\|Cu |
| 3 M $ZnSO_4$ + 10 mM $\alpha$-CD | *J. Am. Chem. Soc.* ***2022**, 144, 11129.* | 99.9% | N.A. | 600 | 1.0 mA $cm^{-2}$/1.0 mAh $cm^{-2}$, Zn\|\|Cu |
| 1 M $Zn(Ac)_2$ + 4 M $NH_4I$ | *J. Am. Chem. Soc.* ***2022**, 144, 18435.* | N.A. | 99.8% | 100 | 1.0 mA $cm^{-2}$/1.0 mAh $cm^{-2}$, Zn\|\|Cu |
| 1 M $Zn(OTf)_2$ in $H_2O$/PC (v/v =1:9) | *J. Am. Chem. Soc.* ***2022**, 144, 7160.* | 99.93% | N.A. | 500 | 1.0 mA $cm^{-2}$/0.5 mAh $cm^{-2}$, Zn\|\|Cu |
| 2 M $ZnSO_4$ + 50 mM DOTf | *Joule* ***2022**, 6, 1103.* | 99.7% | > 99.8% | 3500 | 1.0 mA $cm^{-2}$/0.5 mAh $cm^{-2}$, Zn\|\|Cu |
| 4 m $Zn(BF_4)_2$ in EG/$H_2O$ (w/w = 3:1) | *Nat. Sustain.* ***2022**, 5, 205.* | 99.4% | 99.5 | 400 | 1.0 mA $cm^{-2}$/0.5 mAh $cm^{-2}$, Zn\|\|Cu |

[a] The CE value averaged over the whole cycle life.

[b] The value that CE of individual cycle can stably approach after an initial cycling period.

**Table S4.** Parameters for MD simulations of 10 investigated electrolytes.

| | **$M_3$** | **$M_2$** | **$M_1$** | **M** |
|---|---|---|---|---|
| Number of $Zn^{2+}$ per box | 100 | 100 | 100 | 100 |
| Number of $EMIM^+$ per box | 200 | 200 | 200 | 200 |
| Number of $TFSI^-$ per box | 400 | 400 | 400 | 400 |
| $n$ of $H_2O$ per box | 1000 | 800 | 600 | 400 |
| Total number of atoms | 14100 | 13300 | 12500 | 11700 |
| Simulation box size (Å$^3$) | 54.5×54.5×54.5 | 56.3×56.3×56.3 | 54.5×54.5×54.5 | 54.3×54.3×54.3 |
| | **$M_4$** | **$M_5$** | **$M_6$** | **$M_7$** |
| Number of $Zn^{2+}$ per box | 70 | 40 | 20 | 10 |
| Number of $EMIM^+$ per box | 280 | 320 | 320 | 320 |
| Number of $TFSI^-$ per box | 420 | 400 | 360 | 340 |
| $n$ of $H_2O$ per box | 280 | 160 | 80 | 40 |
| Total number of atoms | 13090 | 13080 | 12140 | 11670 |
| Simulation box size (Å$^3$) | 54.3×54.3×54.3 | 54.3×54.3×54.3 | 55.3×55.3×55.3 | 54.5×54.5×54.5 |
| | **O** | **N** | | |
| Number of $Zn^{2+}$ per box | 72 | 27 | | |
| Number of $Li^+$ per box | | 540 | | |
| Number of $TFSI^-$ per box | 144 | 594 | | |
| Number of $H_2O$ per box | 4000 | 1500 | | |
| Total number of atoms | 18232 | 15477 | | |
| Simulation box size (Å$^3$) | 54.3×54.3×54.3 | 57.1×57.1×57.1 | | |

## References


1. Zhang W, Zhao Q, Hou Y *et al.* Dynamic interphase–mediated assembly for deep cycling metal batteries. *Sci Adv* 2021; **7**: eabl3752.
2. Wang L, Shu T, Guo S *et al.* Fabricating strongly coupled $V_2O_5$@PEDOT nanobelts/graphene hybrid films with high areal capacitance and facile transferability for transparent solid-state supercapacitors. *Energy Storage Mater* 2020; **27**: 150–8.
3. Zhang Y, Wan G, Lewis NHC *et al.* Water or Anion? Uncovering the $Zn^{2+}$ solvation environment in mixed $Zn(TFSI)_2$ and LiTFSI water-in-salt electrolytes. *ACS Energy Lett* 2021; **6**: 3458–63.
4. Yao L, Liu J, Zhang F *et al.* Reconstruction of zinc-metal battery solvation structures operating from −50 ~ +100°C. *Nat Commun* 2024; **15**: 6249.
5. Li P, Roberts BP, Chakravorty DK *et al.* Rational design of particle mesh Ewald compatible Lennard-Jones parameters for +2 metal cations in explicit solvent. *J Chem Theory Comput* 2013; **9**: 2733–48.
6. Ma Y, Zhang Q, Liu L *et al.* N,N-dimethylformamide tailors solvent effect to boost Zn anode reversibility in aqueous electrolyte. *Natl Sci Rev* 2022; **9**: nwac051.
7. Doherty B, Zhong X, Acevedo O. Virtual site OPLS force field for imidazolium-based ionic liquids. *J Phys Chem B* 2018; **122**: 2962–74.
8. Doherty B, Zhong X, Gathiaka S *et al.* Revisiting OPLS force field parameters for ionic liquid simulations. *J Chem Theory Comput* 2017; **13**: 6131–45.
9. Humphrey W, Dalke A, Schulten K. VMD: Visual molecular dynamics. *J Mol Graph* 1996; **14**: 33–8.
10. Wen M, Blau SM, Spotte-Smith EWC *et al.* BonDNet: a graph neural network for the prediction of bond dissociation energies for charged molecules. *Chem Sci* 2021; **12**: 1858–68.
11. Ma G, Miao L, Yuan W *et al.* Non-flammable, dilute, and hydrous organic electrolytes for reversible Zn batteries. *Chem Sci* 2022; **13**: 11320–9.
12. Momma K, Izumi F. VESTA: a three-dimensional visualization system for electronic and structural analysis. *J Appl Crystallogr* 2008; **41**: 653–8.
13. Becker M, Rentsch D, Reber D *et al.* The hydrotropic effect of ionic liquids in water-in-salt electrolytes. *Angew Chem Int Ed* 2021; **60**: 14100–8.

14. Pereiro AB, Araújo JMM, Oliveira FS *et al.* Solubility of inorganic salts in pure ionic liquids. *J Chem Thermodyn* 2012; **55**: 29–36.

15. Wang Y, Li Q, Hong H *et al.* Lean-water hydrogel electrolyte for zinc ion batteries. *Nat Commun* 2023; **14**: 3890.

16. Moumene T, Belarbi EH, Haddad B *et al.* Vibrational spectroscopic study of ionic liquids: Comparison between monocationic and dicationic imidazolium ionic liquids. *J Mol Struct* 2014; **1065–1066**: 86–92.

17. Sun T, Zheng S, Du H *et al.* Synergistic effect of cation and anion for low-temperature aqueous zinc-ion battery. *Nano-Micro Lett* 2021; **13**: 204.

18. Dubouis N, Lemaire P, Mirvaux B *et al.* The role of the hydrogen evolution reaction in the solid–electrolyte interphase formation mechanism for "water-in-salt" electrolytes. *Energy Environ Sci* 2018; **11**: 3491–9.

19. Zhang Q, Ma Y, Lu Y *et al.* Modulating electrolyte structure for ultralow temperature aqueous zinc batteries. *Nat Commun* 2020; **11**: 4463.

20. Huang S, Hou L, Li T *et al.* Antifreezing hydrogel electrolyte with ternary hydrogen bonding for high-performance zinc-ion batteries. *Adv Mater* 2022; **34**: 2110140.

21. Li X, Wang H, Sun X *et al.* Flexible wide-temperature zinc-ion battery enabled by an ethylene glycol-based organohydrogel electrolyte. *ACS Appl Energy Mater* 2021; **4**: 12718–27.

22. Lu Y, Wen Y, Huang F *et al.* Rational design and demonstration of a high-performance flexible $Zn/V_2O_5$ battery with thin-film electrodes and para-polybenzimidazole electrolyte membrane. *Energy Storage Mater* 2020; **27**: 418–25.

23. Wang J, Yang Y, Wang Y *et al.* Working aqueous Zn metal batteries at 100 °C. *ACS Nano* 2022; **16**: 15770–8.

24. Wang N, Yang Y, Qiu X *et al.* Stabilized rechargeable aqueous zinc batteries using ethylene glycol as water blocker. *ChemSusChem* 2020; **13**: 5556–64.

25. Cao C, Zhou K, Du W *et al.* Designing soft solid-like viscoelastic zinc powder anode toward high-performance aqueous zinc-ion batteries. *Adv Energy Mater* 2023; **13**: 2301835.

26. Zhang X, Liu Y, Wang S *et al.* Fundamentals and design strategies of electrolytes for high-temperature zinc-ion batteries. *Energy Storage Mater* 2024; **70**: 103471.

27. Yang T, Xin D, Zhang N *et al.* Interfacial polymerization of PEDOT sheath on $V_2O_5$ nanowires for stable aqueous zinc ion storage. *J Mater Chem A* 2024; **12**: 10137–47.

28. Cong J, Wang Y, Lin X *et al.* Kinetics compensation mechanism in cosolvent electrolyte strategy for aqueous zinc batteries. *J Am Chem Soc* 2025; **147**: 8607–17.

29. Shi X, Zeng J, Yi A *et al.* Unveiling the failure mechanism of zn anodes in zinc trifluorosulfonate electrolyte: the role of micelle-like structures. *J Am Chem Soc* 2024; **146**: 20508–17.

30. Zhao X, Fu J, Chen M *et al.* A self-phase separated electrolyte toward durable and rollover-stable zinc metal batteries. *J Am Chem Soc* 2025; **147**: 2714–25.

31. Wang K, Li Q, Zhang G *et al.* Interface regulation of the Zn anode by using a low concentration electrolyte additive for aqueous Zn batteries. *Chem Sci* 2024; **15**: 230–7.

32. Xu W, Li J, Liao X *et al.* Fluoride-rich, organic–inorganic gradient interphase enabled by sacrificial solvation shells for reversible zinc metal batteries. *J Am Chem Soc* 2023; **145**: 22456−65.

33. Wang Y, Wang Z, Pang WK *et al.* Solvent control of water O−H bonds for highly reversible zinc ion batteries. *Nat Commun* 2023; **14**: 2720.

34. Li C, Kingsbury R, Thind AS *et al.* Enabling selective zinc-ion intercalation by a eutectic electrolyte for practical anodeless zinc batteries. *Nat Commun* 2023; **14**: 3067.

35. Jiang H, Tang L, Fu Y *et al.* Chloride electrolyte enabled practical zinc metal battery with a near-unity Coulombic efficiency. *Nat Sustain* 2023; **6**: 806–15.

36. Han D, Cui C, Zhang K *et al.* A non-flammable hydrous organic electrolyte for sustainable zinc batteries. *Nat Sustain* 2022; **5**: 205–13.

37. Liu M, Yao L, Ji Y *et al.* Nanoscale ultrafine zinc metal anodes for high stability aqueous zinc ion batteries. *Nano Lett*. 2023; **23**(2): 541–9.

38. Dong D, Wang T, Sun Y *et al.* Hydrotropic solubilization of zinc acetates for sustainable aqueous battery electrolytes. *Nat Sustain* 2023; **6**: 1474–84.

39. Zhao K, Fan G, Liu J *et al.* Boosting the kinetics and stability of zn anodes in aqueous electrolytes with supramolecular cyclodextrin additives. *J Am Chem Soc* 2022; **144**: 11129–37.

40. Zhang Q, Ma Y, Lu Y *et al.* Halogenated $Zn^{2+}$ solvation structure for reversible Zn metal batteries. *J Am Chem Soc* 2022; **144**: 18435–43.

41. Ming F, Zhu Y, Huang G *et al.* Co-solvent electrolyte engineering for stable anode-free zinc metal

batteries. *J Am Chem Soc* 2022; **144**: 7160–70.

42. Li C, Shyamsunder A, Hoane AG *et al.* Highly reversible Zn anode with a practical areal capacity enabled by a sustainable electrolyte and superacid interfacial chemistry. *Joule* 2022; **6**: 1103–20.

43. Xiao T, Yang JL, Zhang B *et al.* All‐round ionic liquids for shuttle‐free zinc‐iodine battery. *Angew Chem Int Ed* 2024; **63**: e202318470.

44. Lv Y, Huang C, Zhao M *et al.* Synergistic anion–cation chemistry enables highly stable Zn metal anodes. *J Am Chem Soc* 2025; **147**: 8523–33.

45. Liao X, Chen S, Chen J *et al.* Suppressing Zn pulverization with three- dimensional inert- cation diversion dam for long-life Zn metal batteries. *Proc Natl Acad Sci U S A* 2024; **121**: e2317796121.

46. Yang S, Zhao S, Xu H *et al.* Gradient chaotropic regulation of $Zn^{2+}$ solvation chemistry for low-temperature zinc metal batteries. *Nat Commun* 2026; **17**: 736.
